# Flippases play specific but distinct roles in the development, pathogenicity, and secondary metabolism of *Fusarium graminearum*

**DOI:** 10.1111/mpp.12985

**Published:** 2020-09-02

**Authors:** Yingzi Yun, Pusheng Guo, Jing Zhang, Haixia You, Pingting Guo, Huobin Deng, Yixin Hao, Limei Zhang, Xueyu Wang, Yakubu Saddeeq Abubakar, Jie Zhou, Guodong Lu, Zonghua Wang, Wenhui Zheng

**Affiliations:** ^1^ State Key Laboratory of Ecological Pest Control for Fujian and Taiwan Crops College of Plant Protection Fujian Agriculture and Forestry University Fuzhou China; ^2^ Department of Biochemistry Faculty of Life Sciences Ahmadu Bello University Zaria Nigeria; ^3^ College of Life Science Fujian Agriculture and Forestry University Fuzhou China; ^4^ Institute of Ocean Science Minjiang University Fuzhou China

**Keywords:** DON production, flippase, *Fusarium graminearum*, pathogenicity, secondary metabolism

## Abstract

The membrane trafficking system is important for compartmentalization of the biosynthesis pathway and secretion of deoxynivalenol (DON) mycotoxin (a virulence factor) in *Fusarium graminearum*. Flippases are transmembrane lipid transporters and mediate a number of essential physiological steps of membrane trafficking, including vesicle budding, charging, and protein diffusion within the membrane. However, the roles of flippases in secondary metabolism remain unknown in filamentous fungi. Herein, we identified five flippases (FgDnfA, FgDnfB, FgDnfC1, FgDnfC2, and FgDnfD) in *F. graminearum* and established their specific and redundant functions in the development and pathogenicity of this phytopathogenic fungus. Our results demonstrate that FgDnfA is critical for normal vegetative growth while the other flippases are dispensable. FgDnfA and FgDnfD were found crucial for the fungal pathogenesis, and a remarkable reduction in DON production was observed in Δ*FgDNFA* and Δ*FgDNFD*. Deletion of the *FgDNFB* gene increased DON production to about 30 times that produced by the wild type. Further analysis showed that FgDnfA and FgDnfD have positive roles in the regulation of trichothecene (TRI) genes (*TRI1*, *TRI4*, *TRI5*, *TRI6*, *TRI12*, and *TRI101*) expression and toxisome reorganization, while FgDnfB acts as a negative regulator of DON synthesis. In addition, FgDnfB and FgDnfD have redundant functions in the regulation of phosphatidylcholine transport, and double deletion of *FgDNFB* and *FgDNFD* showed serious defects in fungal development, DON synthesis, and virulence. Collectively, our findings reveal the distinct and specific functions of flippase family members in *F. graminearum* and principally demonstrate that FgDnfA, FgDnfD, and FgDnfB have specific spatiotemporal roles during toxisome biogenesis.

## Graphical abstract

In *Fusarium graminearum*, FgDnfA and FgDnfD positively regulate deoxynivalenol (DON) production, trichothecene gene expressions, and toxisome reorganization, while FgDnfB is a negative regulator of DON synthesis.

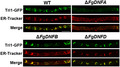

## 1 INTRODUCTION

Fusarium head blight (FHB), caused predominantly by *Fusarium graminearum*, is an economically devastating disease of a wide range of cereal crops, including wheat and barley (Dean *et al*., 2012). This disease not only reduces yield and seed quality, but also poses a great risk to human and animal health owing to its ability to contaminate grains with mycotoxins such as deoxynivalenol (DON), which remains the most frequently detected mycotoxin in contaminated cereal grains (Audenaert *et al*., 2014; Chen *et al*., 2019). The major approach for controlling FHB today is the use of chemical fungicides due to the unavailability of resistant wheat cultivars. Although the application of some commercial fungicides such as azoxystrobin is effective in controlling FHB, the chemicals trigger DON biosynthesis at sublethal concentrations, while others like tebuconazole and the novel cyanoacrylate fungicide phenamacril (JS399‐19) effectively suppress DON production in addition to their FHB‐controlling property (Simpson *et al*., 2001; Magan *et al*., 2002; Chen and Zhou, 2009; Zhang *et al*., 2015). However, there are limited varieties of effective fungicides used in controlling FHB and the available ones are likely to bring a high risk of fungicide resistance (Chen and Zhou, 2009; Yin *et al*., 2009; Willyerd *et al*., 2012). As such, there is the need to uncover more target pathways for developing more effective fungicides and reducing their subsequent resistance by the pathogen. A clear understanding of the key regulatory processes for DON production and for *F. graminearum* pathogenicity is therefore important for efficient management of this disease.

There have been extensive genetics and biochemical studies on the biosynthesis of DON and its derivatives in *Fusarium* (Proctor *et al*., 2018). In *F. graminearum*, the biosynthetic enzymes required for DON production are encoded by 15 *TRI* genes, which are located on different chromosomes, including a gene cluster consisting of 12 core *TRI* genes on chromosome 2, two *TRI* genes (*TRI1* and *TRI16*) on chromosome 1, and a single gene (*TRI101*) on chromosome 3 (Merhej *et al*., 2011; Tang *et al*., 2018). However, only a few studies so far have addressed the cellular processes involved in DON biosynthesis and export in *F. graminearum*. Previous studies in *Penicillium chrysogenum* and *Aspergillus parasiticus* showed that these secondary metabolite (SM)‐producing fungi possess a conserved and compartmentalized SM biosynthetic pathway (Chanda *et al*., 2009; Fernandez‐Aguado *et al*., 2014; Kistler and Broz, 2015). This compartmentalization has also been established recently in *F. graminearum* in relation to DON biosynthesis, where the enzymes involved in DON production have been analysed (Menke *et al*., 2013; Boenisch *et al*., 2017; Tang *et al*., 2018). Menke *et al*. first demonstrated that Tri4 and Tri1 (the proteins involved in the early and late steps of DON biosynthesis) colocalize in a vesicle called a toxisome that is presumed to be the site of trichothecene biosynthesis (Menke *et al*., 2013). Boenisch *et al*. found that growing *F. graminearum* in trichothecene biosynthesis induction (TBI) medium reorganizes the fungal endoplasmic reticulum (ER) to form perinuclear and peripheral structures, and Tri1 and Tri4 colocalize on these structures, suggesting that toxisomes are formed from the ER (Boenisch *et al*., 2017; Chen *et al*., 2019). Tri12, a major facilitator superfamily (MFS) transporter in *F. graminearum*, localizes to the plasma membrane, vacuole, and small (c.1 μm) motile vesicles in the fungal cells in TBI medium; the motile vesicles containing Tri12 may accumulate DON and transport it to the vacuole for storage or the plasma membrane for export via exocytosis (Menke *et al*., 2012, 2013). Based on the above reports, we hypothesize that in *F. graminearum* the membrane trafficking system is important for DON biosynthesis and secretion.

In *F. graminearum* several components of membrane trafficking systems have been shown to be involved in regulating DON production. Molecular motors, soluble *N*‐ethylmaleimide‐sensitive factor attachment protein receptor (SNARE), and the Rab GTPase proteins play critical and conserved roles in vesicle transport and membrane fusion of eukaryotic cells. Tang *et al*. found that the class I myosin of *F. graminearum*, FgMyo1, interacts with Tri1 and actin, and participates in toxisome formation, and demonstrated that the FgMyo1–actin cytoskeleton interaction plays critical roles in DON biosynthesis (Tang *et al*., 2018). Zheng *et al*. have characterized all the 11 *F. graminearum* Rab GTPase proteins by live‐cell imaging and genetic analyses, and shown that they are involved in DON production (Zheng *et al*., 2015). In another study, the SNARE homolog FgVam7 was found to positively regulate the expression of the DON biosynthesis genes *TRI5*, *TRI6*, and *TRI101*, and subsequently DON production (Zhang *et al*., 2016).

A striking aspect of eukaryotic membranes is the uneven distribution of different kinds of phospholipids (membrane asymmetry) across the bilayer, which is essential for proper architecture of the biological membranes (Graham, 2004; Panatala *et al*., 2015). Flippases are responsible for the formation and adjustment of membrane asymmetry and the proteins responsible for flippase activity are type IV P‐type ATPases (P4‐ATPases) (Lee *et al*., 2015). The human genome contains 14 flippases, and mutations in some flippases result in some genetic disorders (Lee *et al*., 2015). In *Arabidopsis thaliana*, 12 proteins constitute the lipid flippase family, and they are responsible for the plant's adaptation to temperature changes, defence responses, and so on (Nintemann *et al*., 2019). *Saccharomyces cerevisiae* has five flippases that mediate a number of steps in membrane trafficking, including vesicle budding, charging, and protein diffusion within membranes (Pomorski *et al*., 2003; Takeda *et al*., 2014).

Despite poor understanding of the roles of flippases in filamentous fungi, some evidence has shown that the proteins may be critically important for fungal growth and pathogenicity. In *Aspergillus nidulans*, Schultzhaus *et al*. found that the flippase AnDnfD is essential for conidiation, and that AnDnfA and AnDnfB work complementarily in the regulation of growth and phosphatidylserine asymmetry (Schultzhaus *et al*., 2015, 2019). In the opportunistic fungal pathogen *Cryptococcus neoformans*, the flippase Apt1 is involved in stress tolerance, polysaccharide secretion, and virulence (Hu and Kronstad, 2010; Rizzo *et al*., 2014). In the rice blast fungus *Magnaporthe oryzae*, the biological functions of two flippases, MoPde1 and MoApt2, have been characterized, and both proteins are involved in fungal virulence (Balhadere and Talbot, 2001; Gilbert *et al*., 2006). In *F. graminearum* recent studies identified FgDnfB and FgNeo1 (FgDnfD homologs) as flippases, and FgDnfB plays a minor role in fungal vegetative growth, polarity maintenance, and conidiation (Zhang *et al*., 2019), while FgNeo1 is important for asexual/sexual developments and virulence in *F. graminearum* (Li *et al*., 2019), but the remaining family proteins remain unknown. In addition, the roles of flippases in the biosynthesis of secondary metabolites have not been established in filamentous fungi.

In the present study we carried out a BLAST search using the amino acid sequences of the *S. cerevisiae* flippases Dnf1, Dnf2, Drs2, Dnf3, and Neo1 against the *F. graminearum* genome and identified five flippases that were named FgDnfA, FgDnfB, FgDnfC1, FgDnfC2, and FgDnfD. Subsequently, we constructed both single and double gene deletion mutants for the various flippase genes and systematically analysed their functions. Our findings reveal not only the active involvement of the flippases in growth, development, and pathogenesis, but also their distinct regulatory roles in DON biosynthesis of *F. graminearum*.

## 2 RESULTS

### 2.1 FgDnfA is crucial for vegetative growth in *F. graminearum*

We generated five hits (FGSG_08595, FGSG_06743, FGSG_09020, FGSG_00595, and FGSG_05149) from a BLAST search using the amino acid sequences of the *S. cerevisiae* flippase proteins Dnf1, Dnf2, Drs2, Dnf3, and Neo1, respectively, against the *F. graminearum* genome (https://blast.ncbi.nlm.nih.gov/Blast.cgi). To use the canonical format of gene nomenclature, we renamed each of the hits *FgDNFA*, *FgDNFB*, *FgDNFC1*, *FgDNFC2*, and *FgDNFD,* respectively, in accordance with a phylogenic analysis and naming convention in *A. nidulans* (Schultzhaus *et al*., 2015). Our phylogenetic analysis suggests that flippases from *S. cerevisiae* and other filamentous fungi, including *A. nidulans*, *F. graminearum*, *M. oryzae*, and *Neurospora crassa*, could be classified into four subgroups (Figure S1a). Of these, each subgroup contains one *F. graminearum* ortholog except subgroup 3, which has the two FgDnfC members, suggesting that the *FgDNFC* gene has undergone duplication relative to its yeast homolog. To investigate the function of the five flippase genes in *F. graminearum* we used a homologous recombination strategy to generate their respective gene deletion mutants, except for *FgDNFB*, which has been generated from our previous study (Zhang *et al*., 2019). The resulting hygromycin‐resistant transformants were screened by PCR (Table S1) and Southern blot (Figure S1b). In addition, we generated a complemented strain for each of the single‐gene deletion mutants by transforming the full DNA sequences (tagged with green fluorescent protein [GFP] at their C‐termini) of the deleted genes into the protoplasts of the respective mutants.

**FIGURE 1:**
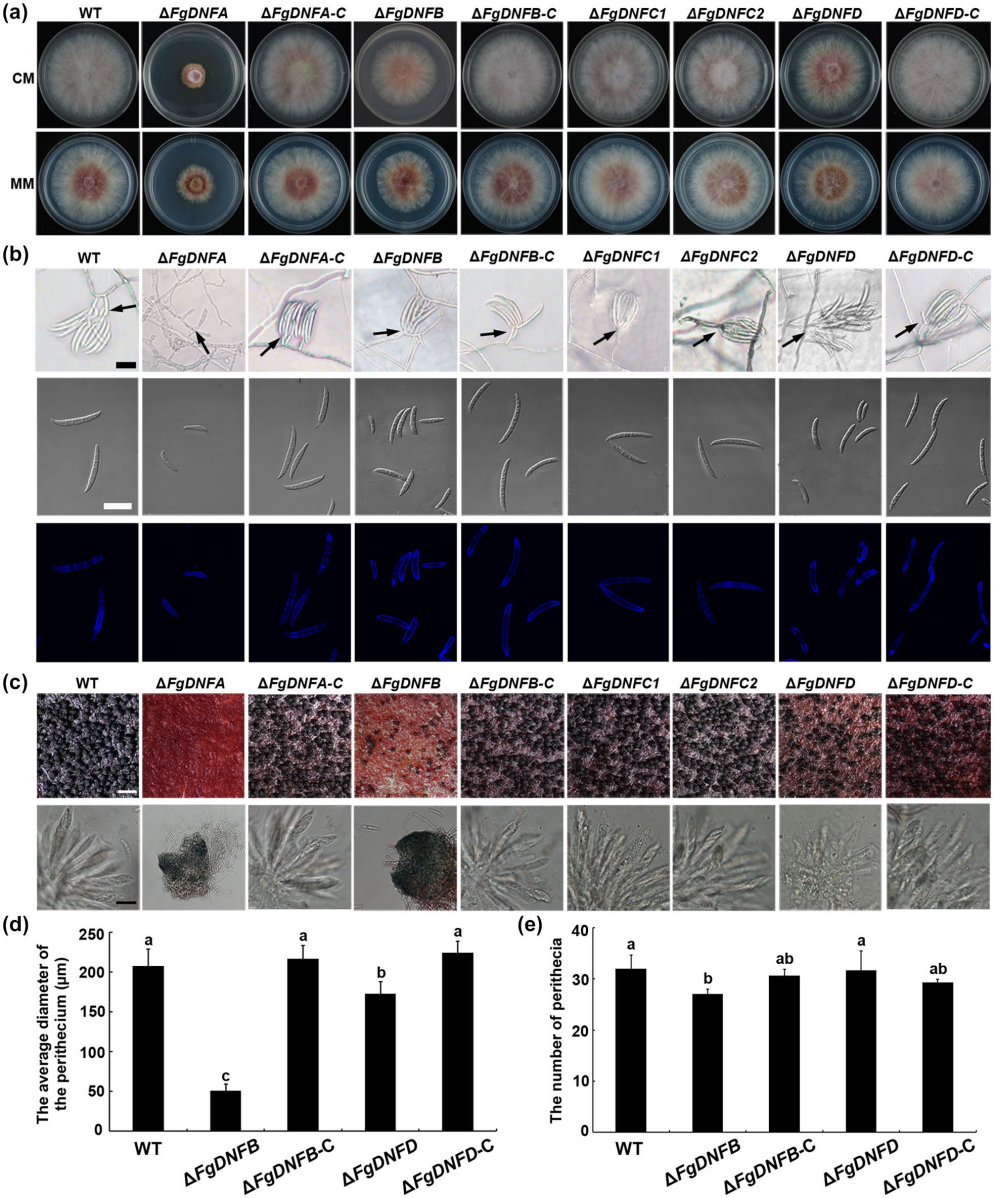
Role of the flippases in the growth and reproduction of *Fusarium graminearum*. (a) Colonies of the wild‐type (WT) strain, Δ*FgDNFA*, Δ*FgDNFB*, Δ*FgDNFC1*, Δ*FgDNFC2*, Δ*FgDNFD*, and complemented strains grown on complete medium (CM) and minimal medium (MM) plates at 28°C for 3 days. (b) Conidiation and conidial morphology (conidiophores indicated by arrows) of the strains on Spezieller Nährstoffarmer agar (SNA) plates. Fresh conidia of the various strains were stained with calcofluor white (CFW). Bar = 30 μm. (c) Perithecia (bar = 200 μm) and ascospores (bar = 10 μm) formation of the indicated strains. (d) Analysis of diameters of perithecia from the indicated strains. (e) The average number of perithecia from the wild‐type, Δ*FgDNFB*, Δ*FgDNFD*, and complemented strains within an area of 360 mm^2^. The same letters on top of the bars indicate insignificant differences at *p* ≥ .05

Of all the five single‐gene deletion mutants generated, only the Δ*FgDNFA* mutant grew significantly more slowly than the wild‐type strain on both complete medium (CM) and minimal medium (MM) (Figure 1a, Table 1). Deletion of *FgDNFB* (Δ*FgDNFB*) also resulted in a slight but insignificant reduction in growth rate compared to the wild‐type strain. However, deletion of the other three flippase genes did not display any clear growth defects on the CM or MM plates (Figure 1a). These results show that among the five flippase proteins in *F. graminearum*, FgDnfA is crucial for vegetative growth of the fungus.

**TABLE 1:** Functional analyses of the wild‐type (PH‐1) , flippase gene deletion mutants, and complemented strains

Strain	Vegetative growth (%)		Conidiation (× 10^6^/ml)	Conidia with different lengths (%)			Conidia with different septa (%)				Virulence^a^	DON production (%)
	CM	MM		≤30 (μm)	30–70 (μm)	≥70 (μm)	0–1	2	3	≥4		
PH‐1	100.0 ± 0.00 A	100.0 ± 0.00 AB	1.32 ± 0.06 A	3	85	12	12	21	48	19	4^a^	100.0 ± 0.00 C
Δ*FgDNFA*	21.5 ± 0.45 D	44.1 ± 2.22 D	0.03 ± 0.02 E	8	92	0	49	5	46	0	1	1.6 ± 0.29 D
Δ*FgDNFA‐*C	99.3 ± 0.64 A	95.0 ± 0.61 B	1.15 ± 0.11 ABCD	2	64	34	7	9	54	30	4	105.2 ± 1.94 C
Δ*FgDNFAC1*	21.7 ± 0.45 D	42.9 ± 0.94 D	0.02 ± 0.01 E	21	79	0	15	20	61	4	1	4.2 ± 0.30 D
Δ*FgDNFAC2*	20.0 ± 2.00 D	43.3 ± 2.40 D	0.04 ± 0.03 E	13	87	0	31	17	51	1	1	4.1 ± 0.11 D
Δ*FgDNFB*	70.8 ± 2.25 C	71.6 ± 0.96 C	0.95 ± 0.11 CD	0	92	8	28	31	40	1	4	3,276.7 ± 583.53 A
Δ*FgDNFB‐*C	100.7 ± 0.65A	104.6 ± 1.96 AB	1.29 ± 0.15 AB	4	68	29	6	9	51	34	4	100.5 ± 0.5 C
Δ*FgDNFBC1*	68.2 ± 0.77 C	68.5 ± 0.35 C	0.92 ± 0.19 D	0	96	4	20	31	45	4	4	4,096.9 ± 309.75 A
Δ*FgDNFBC2*	68.3 ± 1.15 C	66.7 ± 2.86 C	1.10 ± 0.02 ABCD	2	91	7	21	31	46	2	4	3,401.0 ± 433.82 A
Δ*FgDNFBD*	2.0 ± 0.57 E	0 E	0	N/A	N/A	N/A	N/A	N/A	N/A	N/A	0	16.8 ± 9.14 D
Δ*FgDNFD*	84.8 ± 0.67 B	93.5 ± 0.67 AB	1.10 ± 0.2 ABCD	4	87	9	32	26	34	8	2	12.5 ± 5.40 D
Δ*FgDNFD‐*C	100.7 ± 0.65 A	95.8 ± 2.44 B	1.24 ± 0.10 ABC	1	75	24	5	18	57	20	4	101.2 ± 0.20 C
Δ*FgDNFC1D*	83.3 ± 1.22 B	97.7 ± 0.33 B	0.99 ± 0.14 BCD	9	88	3	48	12	31	9	2	5.0 ± 2.80 D
Δ*FgDNFC2D*	88.9 ± 1.05 B	95.4 ± 0.28 B	1.17 ± 0.07 ABCD	16	83	1	62	14	22	2	2	29.9 ± 0.18 D
Δ*FgDNFC1*	101.5 ± 0.66 A	99.6 ± 0.66 B	1.17 ± 0.14 ABCD	3	82	15	6	15	58	21	4	141.4 ± 35.72 B
Δ*FgDNFC2*	99.6 ± 0.63 A	101.1 ± 1.16 AB	1.33 ± 0.10 A	2	65	33	15	12	54	19	4	85.5 ± 24.14 C
Δ*FgDNFC1C2*	96.7 ± 1.14 A	103.4 ± 0.66 A	1.16 ± 0.09 ABCD	0	68	32	6	5	56	33	4	90.4 ± 35.98 C

N/A, not applicable. Single deletions: Δ*FgDNFA*, *B*, *C1*, *C2*, *D*; double deletions: Δ*FgDNFAC1*, *AC2*, *BC1*, *BC2*, *BD*, *C1C2*, *C1D*, *C2D*; complemeted strains: ‐C.

Data followed by same letters indicate insignificant differences at *p* ≥ .01.

### 2.2 FgDnfA and FgDnfB are important for cell membrane‐associated stress response in *F. graminearum*

Because previous studies have shown that flippases may be involved in phosphatidylserine asymmetry, and that phosphatidylserine is a core component of the cell membrane and is necessary for sensing environmental changes (Hankins *et al*., 2015; Schultzhaus *et al*., 2015), we decided to investigate the growth of the flippase gene deletion mutants and their corresponding complemented stains under cell membrane stress conditions. From these assays, we found that both Δ*FgDNFA* and Δ*FgDNFB* mutants showed increased tolerance to membrane stress due to sodium chloride (NaCl), Congo red (CR), and calcofluor white (CFW) compared to the wild‐type strain and the other flippase gene deletion mutants (Figure S2). In addition, the Δ*FgDNFA* mutant showed higher tolerance to sodium dodecyl sulphate (SDS)‐induced stress (Figure S2). These results suggest that FgDnfA and FgDnfB are important for cell membrane‐associated stress responses in *F. graminearum*.

### 2.3 FgDnfA, FgDnfB, and FgDnfD play specific roles in regulating sexual and asexual reproductions in *F. graminearum*

To investigate the roles of the flippases in fungal reproduction, we tested conidiation and perithecia formation of the mutants as compared to the wild‐type and complemented strains. Again, we found that only Δ*FgDNFA* produced a significantly lower amount of conidia (0.03 × 10^6^ conidia/ml) than the wild‐type strain (1.32 × 10^6^ conidia/ml) in carboxymethylcellulose (CMC) medium (Table 1), while deletion of the other four flippase genes did not affect the number of conidia of the mutants when compared to the wild‐type (Table 1). *F. graminearum* conidia are formed in clusters on bottle‐shaped phialides or singly formed on short hyphal branches where the latter style is less efficient than the former (Wang *et al*., 2011; Chen *et al*., 2016). In this study, we tracked the conidiogenesis in each mutant on Spezieller Nährstoffarmer agar (SNA) and found that Δ*FgDNFA* was unable to produce clustered conidia on phialides but formed conidia directly on short hyphal branches, which could account for the reduced conidiation in Δ*FgDNFA* (Figure 1b). We checked the morphology of the conidia obtained from the various strains and found that the conidia produced by Δ*FgDNFA,* Δ*FgDNFB*, and Δ*FgDNFD* were smaller, with fewer septa than the wild‐type and complemented strains (Figure 1b and Table 1), suggesting that the flippases FgDnfA, FgDnfB, and FgDnfD regulate conidial morphology. The germination ability of the conidia from the various strains was tested in 2% sucrose water. The conidial germination of Δ*FgDNFA* was delayed when compared to the wild‐type and the complemented strains (Figure S3), indicating that FgDnfA plays important roles not only in conidiation and conidial morphology, but also in temporal conidial germination in *F. graminearum*.

To investigate the roles of the flippases in the sexual reproduction of *F. graminearum*, the wild‐type, mutants, and complemented strains were grown on carrot medium plates under black‐light conditions to induce sexual reproduction, which is evident by perithecia formation. The results showed that Δ*FgDNFA* only produced several small, nonascus perihelia on the plates (Figure 1c). Δ*FgDNFB* also produced similar but a bit larger perithecia than Δ*FgDNFA*, but the size and number of these perithecia were still smaller and fewer than the perithecia from the wild‐type strain, and no asci were found in them (Figure 1c,d). Δ*FgDNFD* showed better sexual reproduction ability than Δ*FgDNFA* and Δ*FgDNFB*, but it produced significantly smaller perithecia than the wild‐type and complemented strains (Figure 1c,d). Although Δ*FgDNFD* produced asci, no ascospores were found in the perithecia even after 1 month of induction (Figure 1c). Δ*FgDNFC1* and Δ*FgDNFC2* had similar sexual reproduction ability to the wild‐type strain (Figure 1c). These results indicate that the flippases FgDnfA, FgDnfB, and FgDnfD are all required for normal sexual reproduction of *F. graminearum*, but FgDnfA takes the most important role in this process.

### 2.4 FgDnfA and FgDnfD play crucial roles in the pathogenicity of *F. graminearum*

**FIGURE 2:**
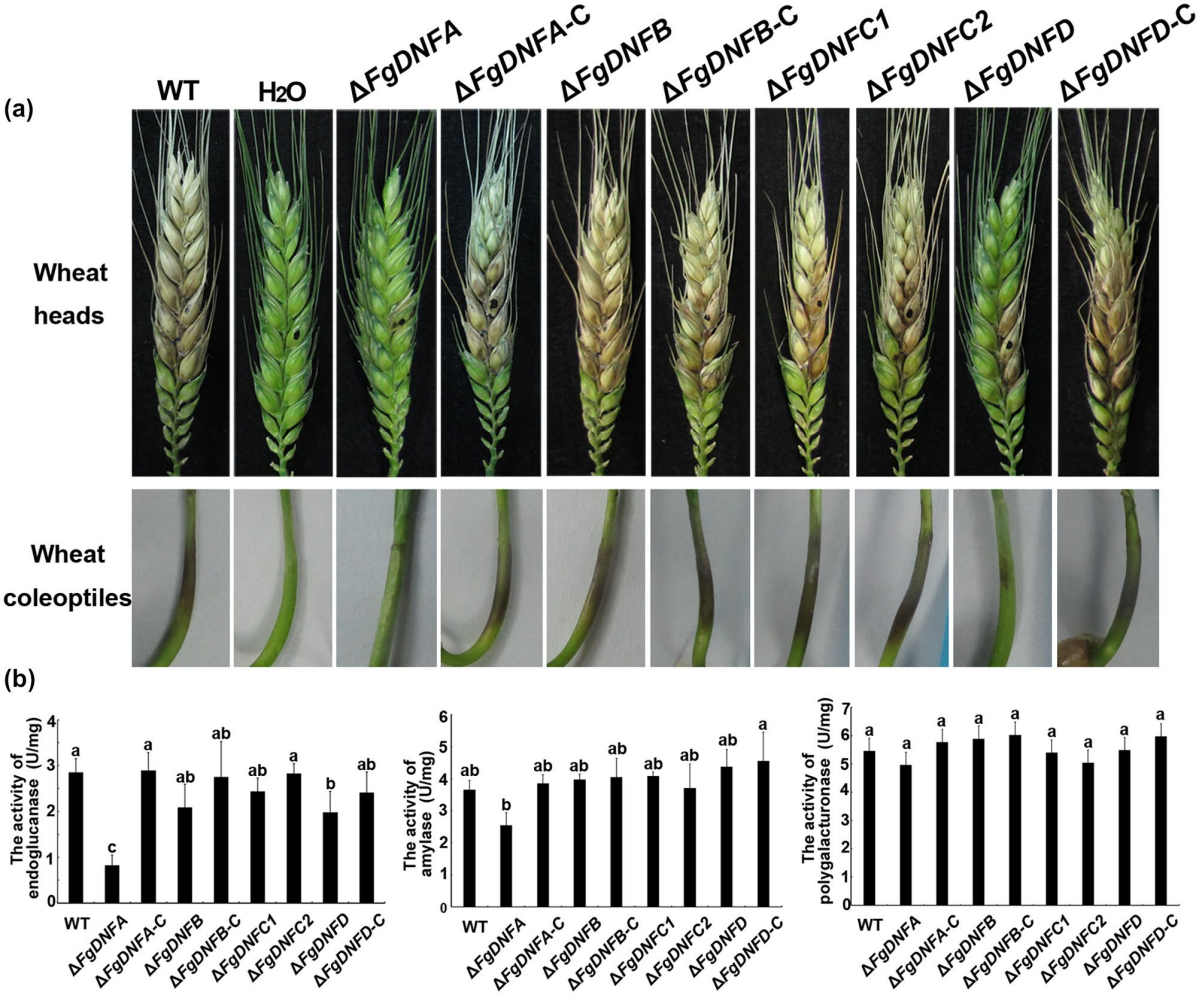
Role of the flippases in the pathogenicity of *Fusarium graminearum*. (a) Comparison of the pathogenicity of the various strains on wheat heads and coleoptiles. Black dots mark the inoculation sites. (b) Activities of three virulence‐related extracellular enzymes, including endoglucanase, amylase, and polygalacturonase in the wild‐type, Δ*FgDNFA*, Δ*FgDNFB*, Δ*FgDNFC1*, Δ*FgDNFC2*, Δ*FgDNFD*, and complemented (‐C) strains after 7 days of induction in Czapek's medium with bran were detected by the 3,5‐dinitrosalicylic acid method. One unit of enzymatic activity is defined as 1 μg/min reducing glucose released from the substrate at pH 4.6 and 50°C

To analyse the roles of the flippases in *F. graminearum* pathogenicity, infection assays on wheat heads and wheat coleoptiles were conducted. As shown in Figure 2a, deletion of *FgDNFA* almost abolished virulence on both wheat heads and wheat coleoptiles. Δ*FgDNFD* appeared more virulent than Δ*FgDNFA*, but the virulence was highly reduced as compared to the wild‐type and complemented strains. The other three flippase gene deletion mutants displayed similar virulence to the wild‐type strain. Similar results were also observed on wheat coleoptiles (Figure 2a), suggesting that FgDnfA and FgDnfD are important for *F. graminearum* pathogenesis.

Extracellular enzymes and DON are important virulence effectors for the pathogenicity of *F. graminearum* (Ma *et al*., 2013), we thus first detected the secreted endoglucanase, amylase, and polygalacturonase activity in wild‐type, flippase mutants, and complemented strains. Our results showed that deletion of *FgDNFA* led to reduced endoglucanase and amylase activities but did not affect the activity of polygalacturonase, while deletion of other flippases did not affect the activities of these three enzymes (Figure 2b). Therefore, these data indicate that the fillipase FgDnfA has a specific role in the regulation of some extracellular enzymes that are responsible for the virulence of *F. graminearum*.

### 2.5 *FgDNFA* and *FgDNFD* positively regulate DON production while *FgDNFB* is a negative regulator in *F. graminearum*

To determine the roles of the flippases in DON biosynthesis, DON production was induced and quantitatively assayed in the wild‐type, mutants, and complemented strains by ELISA. Deletion of *FgDNFA* and *FgDNFD* caused a significant decrease in DON production, where the mutants were able to produce only 1.6% and 12.5% of the DON produced by the wild‐type strain, respectively (Table 1). Surprisingly, deletion of *FgDNFB* increased DON production by about 30 times compared to the wild‐type strain (Table 1). Compared to the wild‐type, deletion of *FgDNFC1* and *FgDNFC2* did not significantly alter the levels of DON production (Table 1). These data indicate that the flippases FgDnfA and FgDnfD positively regulate DON production while FgDnfB acts as a negative regulator.

### 2.6 Deletion of *FgDNFA* or *FgDNFD* suppresses the expression levels of *TRI* genes, while loss of *FgDNFB* has an opposite effect

**FIGURE 3:**
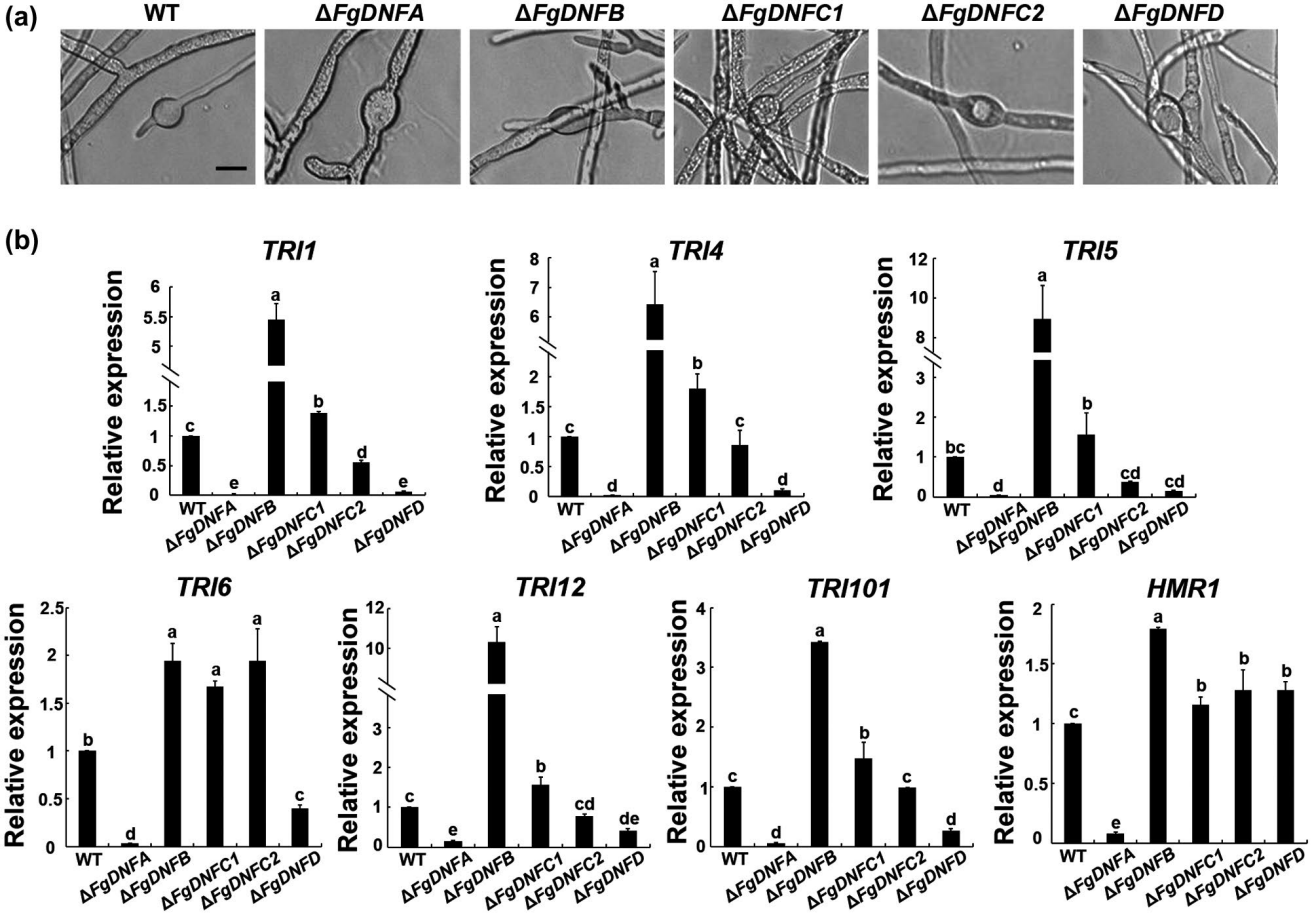
Roles of flippases in the expressions of *TRI* genes. (a) Bulbous structures of the wild‐type (WT) strain, Δ*FgDNFA*, Δ*FgDNFB*, Δ*FgDNFC1*, Δ*FgDNFC2*, and Δ*FgDNFD* in trichothecene biosynthesis induction (TBI) medium after incubation in TBI medium for 1, 2, and 3 days. Bar = 10 μm. (b) Relative expression level of *TRI1*, *TRI4*, *TRI5*, *TRI6*, *TRI12*, *TRI101*, and *HMR1* genes in the indicated strains. The relative expression level of each gene in the Δ*FgDNFA*, Δ*FgDNFB*, Δ*FgDNFC1*, Δ*FgDNFC2*, and Δ*FgDNFD* mutants is the amount of mRNA of each gene relative to the wild‐type strain. The same letters on top of the bars indicate insignificant differences at *p* ≥ .05

Previous work has found that DON production is associated with the formation of intercalary swollen hyphal compartments (Jiang *et al*., 2016), so we observed the mycelial morphology of the strains in DON‐inducing conditions. After 48 hr, hyphal bulbous structures were observed in the mutants relative to the wild type (Figure 3a). No obvious difference was observed with respect to bulbous number and size among the mutants and wild‐type strain, suggesting that the flippase genes are dispensable for normal mycelial structures in DON‐inducing conditions.

To further dissect the mechanisms of the roles played by the flippases in DON production, we evaluated the changes in *TRI* gene expression of the flippase mutants. Our data showed that the expression of six *TRI* genes (*TRI1*, *TRI4*, *TRI5*, *TRI6*, *TRI12*, and *TRI101*) were significantly down‐regulated in the Δ*FgDNFA* and Δ*FgDNFD* mutants, with a more pronounced effect observed in the Δ*FgDNFA* mutant (Figure 3b). In contrast, deletion of *FgDNFB* significantly increased the expression levels of these *TRI* genes. These results are consistent with the DON production assays above. The results further support that FgDnfA and FgDnfD promote DON biosynthesis while FgDnfB is a suppressor for this process.

Hydroxymethylglutaryl (HMG) CoA reductase (Hmr1) is a key enzyme in the isoprenoid biosynthetic pathway for generating farnesyl pyrophosphate, the initial substrate for DON biosynthesis (Boenisch *et al*., 2017). We therefore evaluated the *HMR1* gene expression level in the wild‐type and the flippase mutants. As shown in Figure 3b, the expression level of *HMR1* was obviously down‐regulated (about 13 times less) in Δ*FgDNFA* compared to the wild‐type strain. Although reduction in DON production was recorded in Δ*FgDNFD* mutant, there was no striking change in the expression level of *HMR1* in the mutant when compared to the wild type (Figure 3b), suggesting that FgDnfA and FgDnfD play distinct roles in regulating DON biosynthesis. Similar to the observed effects in *TRI* gene expression, deletion of *FgDNFB* led to an up‐regulation in the expression level of *HMR1*, although the change fold was less than the changes observed in *TRI* gene expressions in the Δ*FgDNFB* mutant (Figure 3b), suggesting that FgDnfB plays a more important role in regulating *TRI* gene expressions than *HMR1* expression. Overall, these results indicate that FgDnfA, FgDnfB, and FgDnfD play critical roles in the DON biosynthesis pathway, but the roles played by FgDnfA and FgDnfD are antagonistic to that played by FgDnfB.

### 2.7 FgDnfA, FgDnfB, and FgDnfD are involved in toxisome biogenesis

**FIGURE 4:**
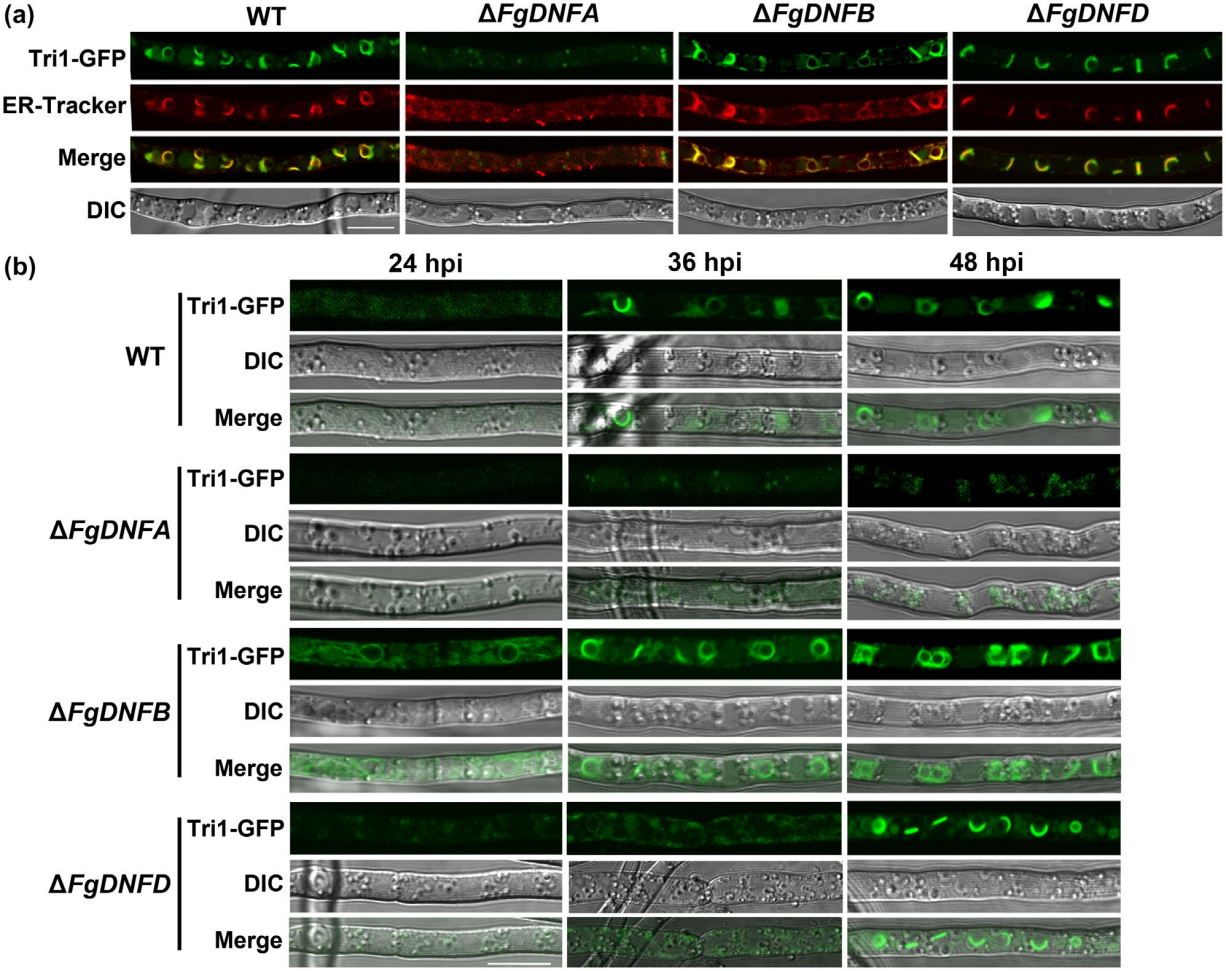
Spatiotemporal roles of FgDnfA, FgDnfB, and FgDnfD during toxisome biogenesis. (a) Tri1‐green fluorescent protein (GFP) fusion protein was expressed in the wild‐type (WT) strain, Δ*FgDNFA*, Δ*FgDNFB*, and Δ*FgDNFD* mutants. The GFP and endoplasmic reticulum (ER)‐tracker signals were observed from the hyphae in the various strains after inoculation in trichothecene biosynthesis induction (TBI) medium at 28°C for 48 hr. Bar = 10 μm. (b) The localization of Tri1‐GFP in the strains was observed after inoculation in TBI medium at 28°C for 24, 36, and 48 hr. Bar = 10 μm

Previous reports demonstrated that Tri4 and Tri1 proteins (which are cytochrome P450 oxygenases) colocalize in some spherical structures called toxisomes, which emanate from reorganized ER during trichothecene induction and are presumed to be the sites for trichothecene biosynthesis (Boenisch *et al*., 2017; Chen *et al*., 2019). We therefore decided to check the subcellular localization of Tri1‐GFP in Δ*FgDNFA,* Δ*FgDNFB*, Δ*FgDNFD*, and the wild‐type strains, respectively. We expressed the Tri1‐GFP construct in the protoplasts of the above strains and, after incubation in TBI medium for 48 hr, the cellular location of FgTri1‐GFP was observed in the transformants of each strain. In the wild‐type strain, the FgTri1‐GFP localized to some spherical and crescent structures in the fungal hyphae, and these structures colocalized with the ER (marked by ER‐specific dye) (Figure 4a), which is consistent with the characteristics of the toxisomes. Although faint signals of FgTri1‐GFP were captured in the Δ*FgDNFA* mutant, the spherical and crescent structures were not observed in this mutant (Figure 4a), suggesting that FgDnfA is essential for the emergence of toxisomes in *F. graminearum*. Despite the fact that Δ*FgDNFD* showed reduced DON production as in Δ*FgDNFA*, loss of *FgDNFD* did not affect the localization of the FgTri1‐GFP in the hyphae after DON induction for 48 hr (Figure 4a), which supports our observation data that FgDnfA plays a more predominant role in DON synthesis than FgDnfD. The appearance of Tri1‐GFP signals in the Δ*FgDNFB* mutant was similar to that in the wild‐type strain after DON induction for 48 hr (Figure 4a) and this is consistent with the negative regulation role of FgDnfB in DON production.

A previous study established that a Tri1‐GFP signal could be visualized after 36 hr of incubation in TBI medium and reached its peak at 48 hr postinoculation (Tang *et al*., 2018). We therefore further observed the Tri1‐GFP signals in the above strains after 24, 36, and 48 hr of incubation in TBI medium (Figure 4b). After 24 hr, no Tri1‐GFP signal was observed in the wild‐type strain, Δ*FgDNFA*, and Δ*FgDNFD*, but obvious Tri1‐GFP signals as well as their spherical appearance were observed in the Δ*FgDNFB* mutant (Figure 4b), indicating that deletion of *FgDNFB* accelerates the accumulation of Tri1 in the cell, which leads to increased DON production in the Δ*FgDNFB* mutant. At 36 hr postincubation, the typical spherical structures with Tri1‐GFP signals were observed in the wild‐type and Δ*FgDNFB* but were not obvious in Δ*FgDNFD* and Δ*FgDNFA* mutants (Figure 4b). However, 48 hr after inoculation, the obvious spherical structures harbouring the Tri1‐GFP signals were observed for the first time in Δ*FgDNFD* but were still inconspicuous in Δ*FgDNFA* (Figure 4b), suggesting the accumulation of Tri1 in the cell is delayed in Δ*FgDNFD*. Put together, the data indicate that FgDnfA, FgDnfB, and FgDnfD play distinct and specific spatiotemporal roles during toxisome biogenesis.

### 2.8 The different flippases have different cellular localizations in *F. graminearum*

**FIGURE 5:**
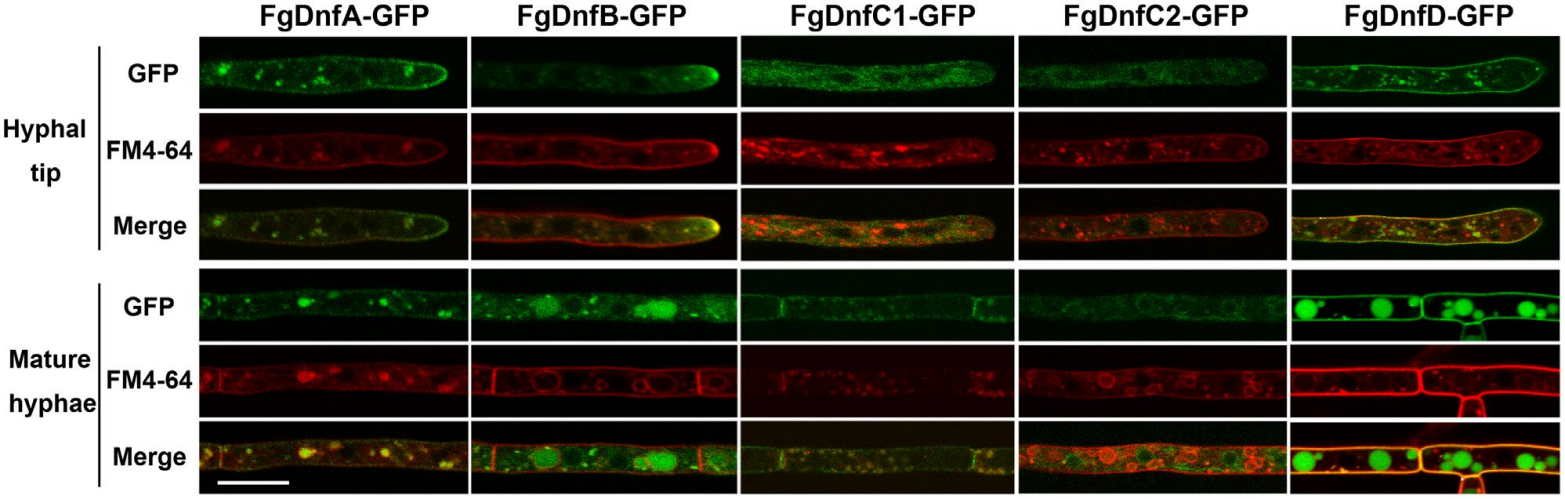
Cellular localization of the flippases in *Fusarium graminearum*. The localizations of the five flippases in the hyphal tip and mature hyphae as well as their colocalizations with the endosome marker FM4‐64 are shown. Bar = 10 μm

Protein domain prediction analysis showed that each flippase in *F. graminearum* has more than seven transmembrane motifs distributed along the protein (Figure S4), which is consistent with their predicted roles as transporters on the membrane. Furthermore, we observed the cellular localization of each flippase in the corresponding complemented strains expressing the respective proteins fused with GFP at their carboxyl termini. As shown in Figure 5, FgDnfA‐GFP mainly localized on the cell membrane, and the FgDnfA‐GFP signals were also observed to colocalize with endosomes stained with FM4‐64 dye, which traces membrane internalization and transport to the vacuolar and endosomal membranes (Zheng *et al*., 2016). However, FgDnfB‐GFP predominantly localized to the Spitzenkörper at the hyphal tip. It is also present at the punctate endosomes in the mature hyphae. FgDnfC1‐GFP and FgDnfC2‐GFP showed relatively weak fluorescent signals and appeared in the cytoplasm of the hyphal tip. However, in mature hyphae FgDnfC1‐GFP is observed in the plasma membrane and septum, while FgDnfC2‐GFP still shows a weak fluorescent signal in the cytosol. FgDnfD‐GFP localized on the cell membrane and colocalized with FM4‐64 at the endosomes in the hyphal tip, and accumulated at the vacuoles in mature hyphae in *F. graminearum*. These results indicate that the flippases FgDnfA, FgDnfB, FgDnfC1, FgDnfC2, and FgDnfD have distinct localizations in *F. graminearum* cells.

Because the FgDnfA, FgDnfB, and FgDnfD colocalize with endosomes, we further investigated the endocytic process in the wild‐type strain and the flippase mutants by staining the strains with FM4‐64 dye and subsequently monitoring the progress of the process after 30 min. However, we did not detect any difference in the internalization of FM4‐64 from the plasma membrane to the vacuoles between these strains and the wild type (Figure S5), indicating that single deletion of the flippases does not affect endocytosis in *F. graminearum*.

### 2.9 Double deletion of *FgDNFC1* or *FgDNFC2* in Δ*FgDNFA*, Δ*FgDNFB*, or Δ*FgDNFD* backgrounds showed no additional phenotypes

To understand whether the flippase genes have functional redundancy in *F. graminearum*, we first analysed the transcription patterns of *FgDNFA, FgDNFB, FgDNFC1, FgDNFC2*, and *FgDNFD* at different developmental stages and during pathogenesis. A heat map was constructed based on previously published RNA‐Seq data during vegetative growth, and sexual and infection processes in *F. graminearum* (Liu *et al*., 2016; Jiang *et al*, 2019). The expression profiles showed that all the five flippases had increased expression levels at the sexual reproduction stage but reduced during pathogenesis (Figure S6a). We further checked the expression levels of the five flippase genes in the fungal tissues growing on CM or DON‐inducing media (TBI) by quantitative reverse transcription PCR (RT‐qPCR). We found that only *FgDNFC1* was significantly up‐regulated by about twofold, whereas the others showed insignificant up‐regulations on TBI as compared to CM medium (Figure S6b). These results suggest that the transcription levels of the five flippase genes at different stages have similar change patterns, which supports functional redundancy of the flippase genes in *F. graminearum*.

To further analyse the functional relationships among the flippase family in *F. graminearum*, we made double gene deletions for the five flippase genes and noticed that FgDnfC1 or FgDnfC2 can be deleted together with *FgDNFA*, *FgDNFB*, or *FgDNFD*, respectively, in a single strain (Table 1). By evaluating the phenotypes of these double‐gene deletion mutants, we found that the mutants showed similar phenotypes to the single‐gene deletion mutants of *FgDNFA*, *FgDNFB*, or *FgDNFD* in vegetative growth, reproduction, virulence, and DON production process (Table 1 and Figure S7). Double‐gene deletion mutants of *FgDNFC1* and *FgDNFC2* also showed similar phenotypes to the wild‐type strain (Table 1 and Figure S7). This indicates that FgDnfC1 and FgDnfC2 are not as important as the other three flippases in mediating the growth and development of *F. graminearum*.

### 2.10 FgDnfB and FgDnfD have redundant functions in growth and development and are involved in phosphatidylcholine transport in *F. graminearum*

**FIGURE 6:**
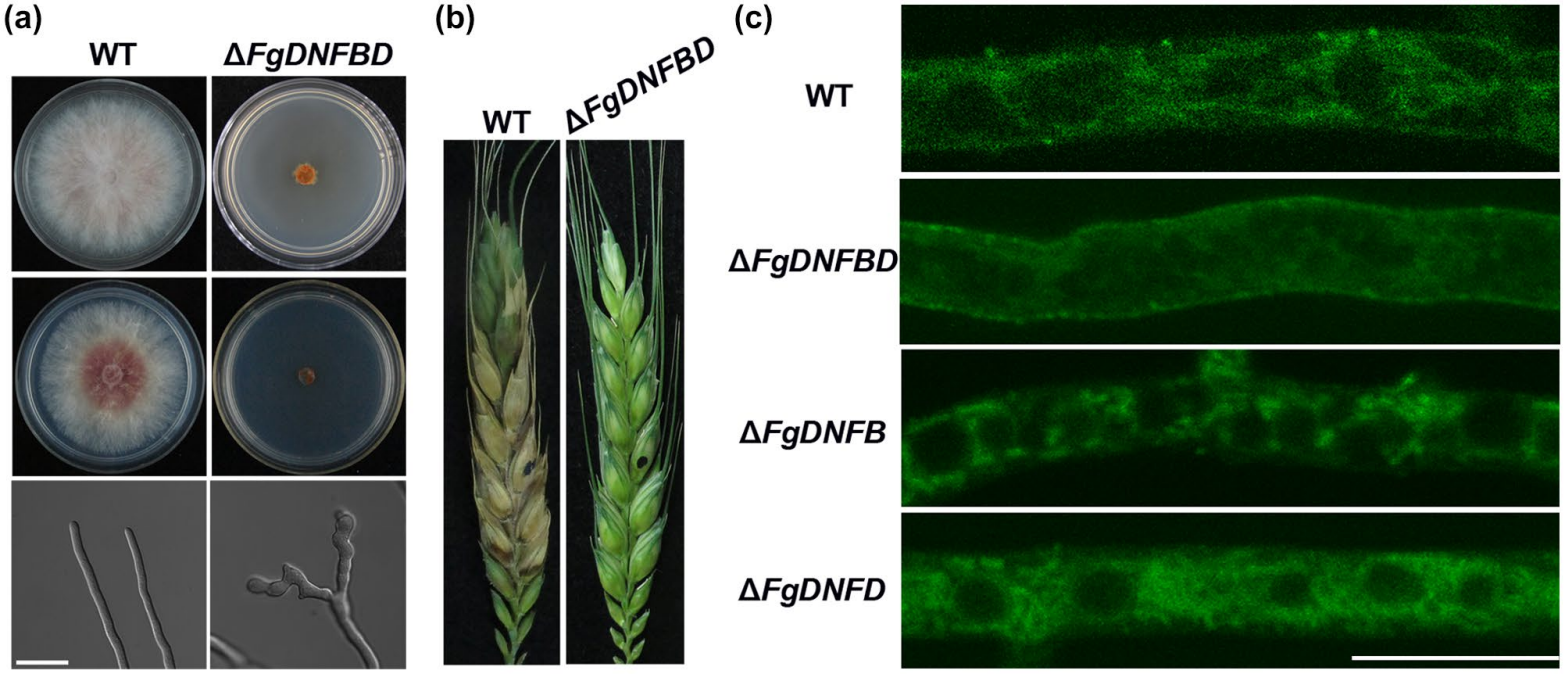
Phenotypic analysis of *FgDNFB*, *FgDNFD*, and *FgDNFBD* mutants. (a) Colonies of the wild‐type (WT) strain and Δ*FgDNFBD* on complete medium (CM) and minimal medium (MM) plates after incubation for 3 days at 28°C. Fresh mycelia on the CM plates were observed under a scanning microscope. Bar = 10 μm. (b) Pathogenicity of the wild‐type strain and Δ*FgDNFBD* mutant on wheat heads. Black dots mark the inoculation sites. (c) The wild‐type strain, Δ*FgDNFB*, Δ*FgDNFD*, and Δ*FgDNFBD* mutants were treated with 7‐nitro‐2‐1,3‐benzoxadiazol‐4‐yl‐phosphatidylserine (NBD‐PS) and observed under a confocal microscope. Bar = 10 μm

Among the five flippase genes in *F. graminearum*, we generated double‐gene deletion mutants in different permutations except for two, *FgDNFA* with *FgDNFB* and *FgDNFA* with *FgDNFD*, which could not be obtained after several screenings (Table 1). We hypothesized that *FgDNFA* cannot be deleted together with either *FgDNFB* or *FgDNFD* in *F. graminearum*. However, we obtained a double deletion mutant for *FgDNFB* and *FgDNFD*; remarkably, the double deletion mutant Δ*FgDNFBD* displayed a serious defect in vegetative growth compared to the wildtype (Figure 6a) and even when compared to the single deletion mutants of the two genes (Figure 1a). We also observed that hyphae from the Δ*FgDNFBD* double mutant were highly branched and curled (Figure 6a). In addition, we found that Δ*FgDNFBD* could not produce conidia and perithecia, and almost lost virulence on wheat heads (Figure 6b). DON production was also highly reduced in this mutant (Table 1). These results suggest that FgDnfB and FgDnfD have redundant functions in regulating vegetative growth, reproduction, and pathogenicity in *F. graminearum*.

Fluorescent phospholipids can be used to analyse the substrate specificity of flippases (Lopez‐Marques *et al*., 2014). Thus, we observed the cellular location of 7‐nitro‐2‐1,3‐benzoxadiazol‐4‐yl (NBD)‐tagged phospholipids in the wild‐type strain and the different flippase deletion mutants. We observed the fluorescence signals in mycelia containing NBD‐labelled phosphatidylcholine (NBD‐PC), phosphatidylserine (NBD‐PS), and phosphatidylethanolamine (NBD‐PE), respectively, and the fluorescence signals were detected from NBD‐PC and NBD‐PS only under a fluorescence microscope. There was a similar NBD‐PS accumulation pattern in all the strains, including the eight double‐gene deletion mutants (Figure S8). Also, NBD‐PC accumulated similarly in the cytoplasm of all the strains except for the Δ*FgDNFBD*, and we found that only Δ*FgDNFBD* showed NBD‐PC signals on the cell membrane (Figures 6c and S9), suggesting that the double‐gene deletion Δ*FgDNFBD* leads to the defect in transporting the NBD‐PC into the cytoplasm. Collectively, these results support the redundant functions of FgDnfB and FgDnfD in growth and development and that the two flippases are involved in phosphatidylcholine transport in *F. graminearum*.

## 3 DISCUSSION

The present study systematically analysed the functions of the five flippases in *F. graminearum* using genetic, biochemical, and cell biology approaches. Despite functional redundancy among the flippases, FgDnfA, FgDnfB, and FgDnfD still have distinct and specific roles in regulating the vegetative growth, reproduction, secondary metabolism, and pathogenicity of *F. graminearum*. To our knowledge, this is the first detailed and comprehensive functional analysis of flippases in a plant‐pathogenic fungus.

Flippases are ATP‐dependent transporters belonging to the P4 subfamily of P‐type ATPases (P4‐ATPases), which are only present in eukaryotic organisms (Daleke, 2007). Among the five flippase genes in *S. cerevisiae*, only *NEO1* is essential for viability; deletion of any one of the other four genes is not lethal, but the quadruple mutant (∆*DRS2*∆*DNF1*∆*DNF2*∆*DNF3*) is not viable, indicating that they have overlapping functions (Daleke, 2007). In filamentous fungi, four flippases have been found in the genome of *A. nidulans* and all of them, including the homolog gene for *NEO1*, are not essential genes (Schultzhaus *et al*., 2015, 2019). However, double‐gene deletion for *AnDNFA* (homolog gene of *DNF1* and *DNF2*) and *AnDNFB* (homolog gene of *DRS2*) or *AnDNFB* and *AnDNFD* (homologue gene of *NEO1*) was lethal for the growth of *A. nidulans*, suggesting that functional redundancy of flippases also exists in *A. nidulans* (Schultzhaus *et al*., 2015, 2019). In this study, five flippase genes were identified in the genome of *F. graminearum*. We also found functional redundancy among these genes. Similar to *A. nidulans*, *FgDNFA* (homolog gene of *DNF1* and *DNF2*) and *FgDNFB* (homolog gene of *DRS2*) could not be double‐deleted in a single fungal cell. In contrast to *A. nidulans*, *FgDNFB* and *FgDNFD* could be double‐deleted in *F. graminearum* and the double‐deletion mutant displayed more drastic defects than the individual single‐deletion mutants, suggesting that *FgDNFB* and *FgDNFD* share overlapping functions (Figure 6). In *F. graminearum*, *FgDNFA* and *FgDNFD* (homolog gene of *NEO1*) could not be double‐deleted, but in *A. nidulans*, double‐deletion mutants for *AnDNFA* and *AnDNFD* showed similar defects as the deletion mutant of *AnDNFA* (Schultzhaus *et al*., 2019). These results indicate functional redundancy that normally exists among flippases in fungi even though the same flippase homologs in different organisms have functional diversity.

Study of the flippases in *A. nidulans* revealed their functions in the regulation of vegetative growth and asexual sporulation (Schultzhaus *et al*., 2015, 2019). Here, we also confirmed the involvement of flippases in growth and conidia production in *F. graminearum* (Figure 1 and Table 1). Additionally, we found that different members of the flippase family play different roles in regulating sexual reproduction in ascomycetes. Sexual reproduction is important in the life cycle of *F. graminearum* and sexual spores (ascospores) from perithecium (the sexual fruiting body) serve as primary inocula for FHB (Dill‐Macky and Jones, 2000; Guenther and Trail, 2005). Most importantly, our data showed that FgDnfA, FgDnfB, and FgDnfD play important roles not only in sexual reproduction, but also in mediating different sexual developmental stages of *F. graminearum*. Li *et al*. constructed a conditional suppress mutant for *FgDNFD* and found that *FgDNFD* is required for ascospore discharge in an ion‐dependent manner (Li *et al*., 2019). However, in this study we found that the deletion mutant of *FgDNFD* produces smaller perithecia than the wild‐type strain and does not produce ascospores (Figure 1), indicating that FgDnfD is essential for maturation of perithecia in *F. graminearum*. A previous work demonstrated that perithecia formation requires lots of energy and precursors, and lipids may be the key resources supporting this cellular process (Lee *et al*., 2011). In the metabolic pathway of the neutral lipid triacylglycerol in *S. cerevisiae*, phosphatidylinositol, including phosphatidylserine, phosphatidylethanolamine, and phosphatidylcholine, are the intermediate products (Wang, 2015). In this study, we found that Δ*FgDNFA* mutant displays suppressed growth on CM but the growth improved markedly on MM (Figure 1a and Table 1). This is similar to the growth pattern of the deletion mutant of Fg10302, a homolog of phosphatase gene (*NEM1*) involved in the triacylglycerol metabolic pathway in *S. cerevisiae* (Yun *et al*., 2015), suggesting that deletion of *FgDNFA* may also affect the metabolism of triacylglycerol. Based on these results, we hypothesize that flippases in *F. graminearum* may be involved in the biosynthesis of phospholipids, which in turn makes them important for sexual reproduction in the phytopathogenic fungus.

In fungal pathogens, flippases are known to be required for effective pathogenesis. Documented reports in the rice blast fungus *M. oryzae*, the opportunistic fungal pathogen *C. neoformans*, and *F. graminearum* suggest that flippases have conserved roles in the regulation of pathogenicity, but different homologs display functional diversity in different fungal pathogens (Balhadere and Talbot, 2001; Gilbert *et al*., 2006; Hu and Kronstad, 2010; Rizzo *et al*., 2014; Li *et al*., 2019). The homologs of Drs2 of *S. cerevisiae* were named as MoPde1 and Apt1 in *M. oryzae* and *C. neoformans*, respectively. MoPde1 is involved in regulating host penetration and hyphal development in *M. oryzae* while loss of Apt1 in *C. neoformans* led to decreased survival of the mutant in the lungs of infected mice and the inability of the mutant to colonize brain tissues, indicating that MoPde1 and Apt1 are required for invasive growth in the respective organisms (Balhadere and Talbot, 2001; Hu and Kronstad, 2010; Rizzo *et al*., 2014). However, loss of FgDnfB, the homolog of Drs2, does not affect the virulence of *F. graminearum* (Figure 2).

We found that FgDnfA and FgDnfD function as positive regulators while FgDnfB takes the negative role in DON biosynthesis, which is the first evidence to establish the relationship between flippases and secondary metabolism in filamentous fungi. Filamentous fungi produce a diverse range of secondary metabolites including mycotoxins, which have negative impacts on food safety and animal health but which are potentially important for fungal pathogenesis (Kistler and Broz, 2015). In *A. nidulans*, deletion of *AnDNFA* or *AnDNFD* resulted in mutants that produce unpigmented conidia (Schultzhaus *et al*., 2015, 2019). With the exception of these reports, there was no work that relates the functions of flippases to secondary metabolism. Research on the three well‐studied fungal secondary metabolite biosynthetic pathways (penicillin G, aflatoxin, and DON synthesis pathways) showed that co‐compartmentalization of secondary metabolism enzymes is important in promoting pathway efficiency and sequestering intermediates and products from the rest of the cell (Chanda *et al*., 2009; Kistler and Broz, 2015; Boenisch *et al*., 2017). For DON biosynthesis, formation of the toxisome from ER is necessary to ensure compartmentalization of the process (Chen *et al*., 2019). In this study, we found that deletion of the flippases *FgDNFA*, *FgDNFB*, and *FgDNFD* (most especially *FgDNFA*) affects the normal biogenesis of toxisomes in DON‐inducing medium (Figure 4), suggesting that the flippases are required for establishing compartmentalization of the secondary metabolism pathway. In addition, deletion of these flippases perturbs the expression of *TRI* genes, which is consistent with the observed changes in DON production of the respective mutants. It is worth noting that FgDnfB acts as a negative regulator of DON biosynthesis, unlike FgDnfA and FgDnfD, which positively regulate the process. Because the endosome serves as a central hub for signal communication and protein trafficking, and considering that FgDnfA, FgDnfD, and FgDnfB localize to the endosomal membrane, the flippases are probably involved in coordinating endosomal membrane‐dependent compartmentalization, but the underlying mechanisms require further investigation.

The specific functions of FgDnfA, FgDnfB, and FgDnfD are reflections of their regulatory roles in *F. graminearum* sexual reproduction and DON synthesis. As such, we hypothesize that their specific functions have close relationships with their respective substrate specificity. However, most of our current knowledge about the functions and substrate specificity of flippases in fungi have been derived from previous studies in budding yeasts, despite the fact that budding yeasts are in many ways different from filamentous fungi in terms of cellular metabolism. In *A. nidulans*, AnDnfA and AnDnfB were demonstrated to be responsible for the transport of phosphatidylserine (Schultzhaus *et al*., 2015). Herein, we found that FgDnfB and FgDnfD are needed for the transport of phosphatidylcholine. However, substrate specificity is still rarely understood in filamentous fungi. Functional redundancy among flippases aggravates the difficulty in analysing the substrate of a flippase. For this reason, multiple gene deletions and additional biochemical analyses should be integrated for more effective evaluation of the functional mechanisms of flippases in filamentous fungi.

## 4 EXPERIMENTAL PROCEDURES

### 4.1 Strains and culture conditions

The wild‐type strain PH‐1 and all transformants used in this study were stored as mycelial suspensions in 20% glycerol solution at −80°C. CM and MM were used for mycelial growth tests. CMC medium and SNA were used for conidiation assays (Leslie and Summerell, 2006). For conidial germination, fresh mycelial plugs of each strain were inoculated on SNA plates at 28°C for 7 days. Sexual reproduction was induced on carrot medium as described previously (Zheng *et al*., 2015). TBI medium was used for the induction of DON (Menke *et al*., 2012).

### 4.2 Strain construction

For construction of gene deletion mutants, the upstream and downstream fragments of the target gene were amplified by the primers listed in Table S1. Double‐joint PCR was used to build a gene replacement construct (Yu *et al*., 2004), which was transformed into the protoplasts of the wild‐type strain to generate the gene deletion mutants (Hou *et al*., 2002). The resulting transformants were screened by PCR using the primers shown in Table S1 and further verified by Southern blot. Hygromycin (100 mg/ml) or geneticin (150 mg/ml) was used as a selective marker for single‐ or double‐gene deletion, respectively. For complementation, the entire target gene (without stop codon), including its promoter region, was amplified by PCR using the set of primers listed in Table S1 and transformed with *Xho*I‐digested pYF11 using the yeast gap repair approach (Zhou *et al*., 2011). The resulting target gene with a GFP fusion construct carrying the geneticin resistance gene was introduced into the corresponding mutant's protoplasts, and the resulting transformants were selected in geneticin (150 mg/ml)‐containing media. For observation of the Tri1‐GFP location, the generated FgTri1‐GFP fusion vector (Adnan *et al*., 2020) was transformed into the wild type and the corresponding mutants’ protoplasts and geneticin (150 mg/ml) was used as a selective marker.

### 4.3 Pathogenicity and DON production assays

Pathogenicity assays on wheat spikelets were conducted as described previously (Yun *et al*., 2014). In brief, 10 μl of conidia suspension (10^6^ conidia/ml) or a mycelial block (3 mm in diameter) of each strain was inoculated in the middle of spikelets of wheat flowers, and then the inoculated wheat head was covered with a plastic bag to keep it humid for 2 days. It was observed 2 weeks after inoculation. For wheat coleoptile infection assays, 10 μl of conidial suspensions (4 × 10^5^ conidia/ml) were inoculated and symptoms were observed 8 days after inoculation. For DON production assays, each strain was grown in TBI at 28°C for 7 days in the dark. The liquid and mycelia were then collected. The liquid solution was tested quantitatively for DON using a Vomitoxin ELISA kit (Finder Biotech Co.) (Zheng *et al*., 2018), while the mycelia were dried and weighed for quantification. Each experiment was repeated three times.

### 4.4 Measurement of extracellular enzyme activity

For detection of the activity of extracellular enzymes, three fresh mycelial plugs (5 mm in diameter) from each strain were inoculated in a 250‐ml flask containing 100 ml of Czapek's medium at 25°C for 7 days. Mycelia were completely removed by filtration, and the culture filtrates were used for the measurement of extracellular enzyme activities. The activities of endoglucanase, amylase, and polygalacturonase were determined using the 3,5‐dinitrosalicylic acid method with slight modifications, as previously described (Miller, 1959). The dry weights of the harvested mycelia were measured for normalizing the enzyme activities.

### 4.5 Live‐cell imaging assay

Fresh conidia were collected after 4 days of incubation in CMC medium and then stained with 10 μg/ml CFW for 2 min. The cell walls and septa of the conidia were observed under an A1 confocal microscope (Nikon). Fresh mycelia of each strain were stained with 2 μM FM4‐64 and observed for endocytosis under an A1 confocal microscope. ER‐Tracker Red (Beyotime Biotechnology) was used to label the ER. NBD‐PS/PC/PE (Avanti Polar Lipids) was used for phospholipid staining as previously described (Hanson and Nichols, 2001). In brief, young mycelia were suspended in ice‐cold MM‐S medium (MM medium without sucrose but with 2% sorbitol) with 10 μl of lipid dye and incubated at 30°C for 30 min, then washed with ice‐cold MM‐S and observed under an A1 confocal microscope. The wavelengths of excitation/emission used for NBD‐PS/PC/PE were 488 nm/500–550 nm.

### 4.6 RT‐qPCR analysis

Total RNA of each strain was isolated from mycelia harvested from 3‐day‐old TBI cultures or 3‐day‐old CM cultures using TRIzol. To detect the relative expression levels of the target genes, SYBR Premix Ex Taq II (Takara) was used for RT‐qPCR. The tubulin gene (FGSG_09530) of *F. graminearum* was used as the endogenous control, and the relative expression levels of the target genes were calculated using 2^−ΔΔ^ *^C^* ^t^ formula (Livak and Schmittgen, 2001). The experiments were repeated three times.

## Supporting information


**FIGURE S1** Identifications and deletions of the flippase genes in *Fusarium graminearum*. (a) Phylogenic analyses of the *F. graminearum* flippases FgDnfA, FgDnfB, FgDnfC1, FgDnfC2, and FgDnfD with their orthologs in other fungi, including *Saccharomyces cerevisiae* (ScDnf1, ScDnf2, ScDnf3, ScDrs2, and ScNeo1), *Neurospora crassa* (NcDnfA, NcDnfB, NcDnfC1, NcDnfC2, and NcDnfD), *Aspergillus* *nidulans* (AnDnfA, AnDnfB, AnDnfC, and AnDnfD), and *Magnaporthe oryzae* (MoPde1, MoApt2, MoApt3, MoApt4, and MoApt5) using the neighbour‐joining method from MEGA 7 software. Values on clusters branches represent the results of bootstrap analysis. (b) Southern blot hybridization analysis of the indicated mutants using hygromycin DNA fragment (*HPH*) as a probe

**FIGURE S2** Environmental stress responses of the flippase mutants in* Fusarium graminearum*. (a) Colonies of each strain growing on CM medium containing 0.01% SDS, 0.8 M NaCl, 12 mM H_2_O_2_, 0.5 mg/ml Congo red (CR) or 250 μg/ml calcofluor white (CFW). (b) Statistical analysis of mycelial growth inhibition due to the indicated stress‐inducing agents. Error bars represent *SD* from three replicates and the same letters on top of the bars indicate insignificant differences at *p* ≥ .05

**FIGURE S3** Conidial germination rates of the flippase mutants. Fresh conidia were inoculated in CM liquid for 1, 2, 3, and 4 hr, and the germination of 50 conidia from the indicated strain was observed, respectively. The same letters on top of the bars indicate insignificant difference at *p* ≥ .05

**FIGURE S4** Protein structure of the different flippases in *Fusarium graminearum*. The structural representations of the five flippase proteins in *F. graminearum* are shown. Blue represents the transmembrane region, pink represents the low complexity region, and green stands indicates the coiled coil region

**FIGURE S5** Analyses of endocytosis in the flippase mutants of *Fusarium graminearum*. FM4‐64 dye internalization in the wild‐type, Δ*FgDNFA*, Δ*FgDNFB*, Δ*FgDNFC1*, Δ*FgDNFC2*, and Δ*FgDNFD* was observed after 5 and 30 min. Bar = 10 μm

**FIGURE S6** Relative gene expression levels of the five flippase genes in *Fusarium graminearum*. (a) The expression profiles of the five flippase genes at different stages of *F. graminearum* development: vegetative growth (potato dextrose agar), sexual induction for 3 days (S 3d) and 8 days (S 8d), and infection assay on wheat heads at 1, 2, and 3 days postinoculation (I 1d, I 2d, and I 3d). Each flippase gene was up‐regulated during the sexual process but down‐regulated during the infection process compared to the normal vegetative growth. (b) The relative expression levels of the flippase genes in the fungal mycelia grown on TBI compared to CM medium. Error bars represent *SD* from three replicates and the same letters on top of the bars indicate insignificant differences at *p* ≥ .05

**FIGURE S7** Functional analyses of flippase double deletion mutants. (a) Colonies of the wild‐type strain, Δ*FgDNFAC1*, Δ*FgDNFAC2*, Δ*FgDNFBC1*, Δ*FgDNFBC2*, Δ*FgDNFDC1*, Δ*FgDNFDC2*, and Δ*FgDNFDC1C2* mutants on complete medium (CM) and minimal medium (MM) plates after incubation for 3 days at 28 °C. (b) Fresh conidia from the indicated strains stained with CFW. Bar = 30 μm. (c) Perithecia (bar = 200 μm) and ascospores (bar = 10 μm) formation. (d) Pathogenicity of the indicated strains on wheat heads

**FIGURE S8** Cellular localization of NBD‐PS in flippase mutants of *Fusarium graminearum*. The wild‐type strain, flippase single and double gene deletion mutants were treated with NBD‐PS and observed under a confocal microscope. Bar = 10 μm

**FIGURE S9** Cellular localization of NBD‐PC in flippase mutants of *Fusarium graminearum*. The wild‐type strain, flippase single and double gene deletion mutants were treated with NBD‐PC and observed under a confocal microscope. Bar = 10 μm

**TABLE S1** The primers used in this study

## Data Availability

The data that support the findings of this study are available from the corresponding author upon reasonable request.

## Footnotes

1 Virulence on wheat heads was scored (4, normal; 0, markedly reduced virulence compared to wild‐type strain).
