# Supplementary material for: Flippases play specific but distinct roles in the development, pathogenicity, and secondary metabolism of Fusarium graminearum
Source: Mol Plant Pathol. 2020 Sep 2;21(10):1307–21. doi: 10.1111/mpp.12985 (PMC7488471; doi:10.1111/mpp.12985)
Supplement: Supplementary file 1 — FIGURE S1 Identifications and deletions of the flippase genes in Fusarium graminearum. (a) Phylogenic analyses of the F. graminearum flippases FgDnfA, FgDnfB, FgDnfC1, FgDnfC2, and FgDnfD with their orthologs in other fungi, including Saccharomyces cerevisiae (ScDnf1, ScDnf2, ScDnf3, ScDrs2, and ScNeo1), Neurospora crassa (NcDnfA, NcDnfB, NcDnfC1, NcDnfC2, and NcDnfD), Aspergillus nidulans (AnDnfA, AnDnfB, AnDnfC, and AnDnfD), and Magnaporthe oryzae (MoPde1, MoApt2, MoApt3, MoApt4, and MoApt5) using the neighbour‐joining method from MEGA 7 software. Values on clusters branches represent the results of bootstrap analysis. (b) Southern blot hybridization analysis of the indicated mutants using hygromycin DNA fragment (HPH) as a probe [file MPP-21-1307-s001.docx]

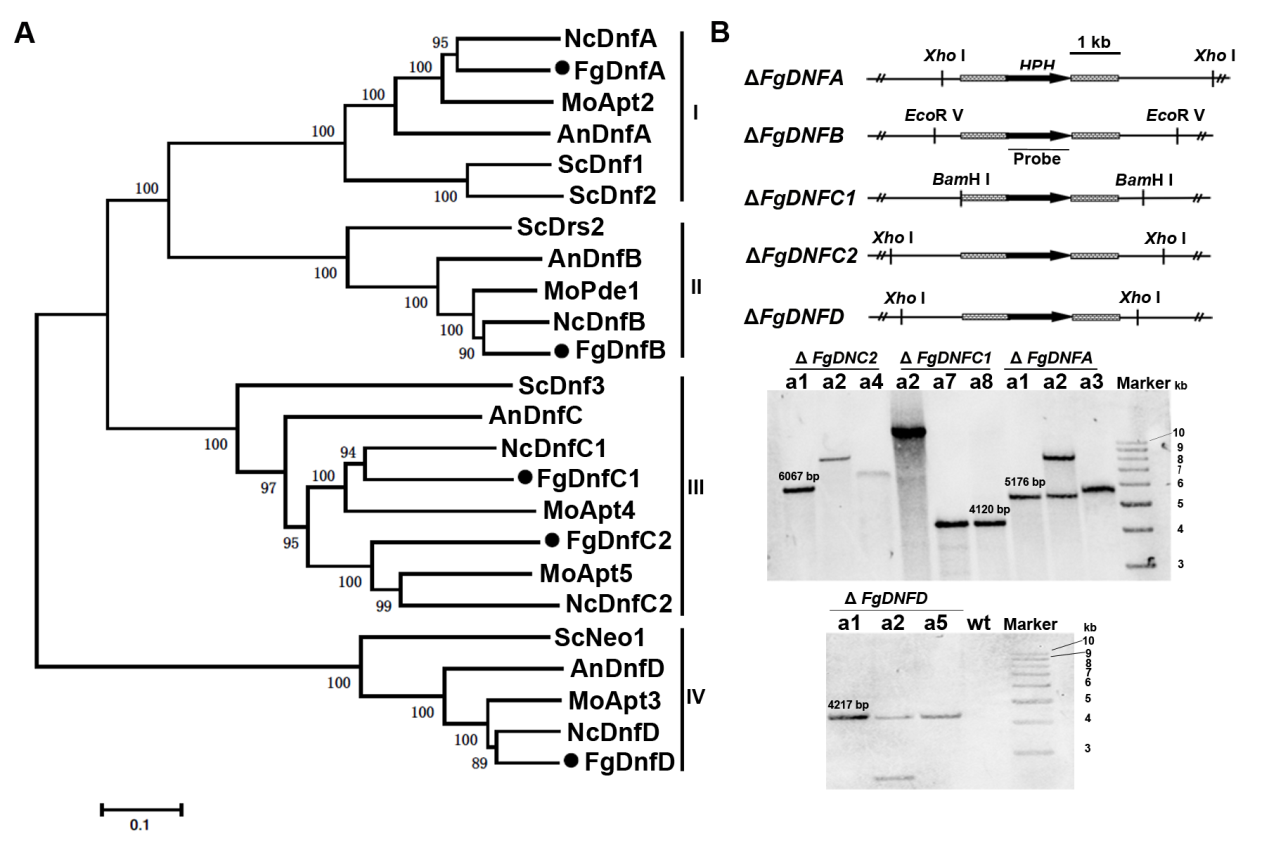


**Fig. S1 Identifications and deletions of the flippase genes in *F. graminearum***

(A) Phylogenic analyses of the *F. graminearum* flippases FgDnfA, FgDnfB, FgDnfC1, FgDnfC2 and FgDnfD with their orthologs in other fungi including *Saccharomyces cerevisiae* (ScDnf1, ScDnf2, ScDnf3, ScDrs2, and ScNeo1), *Neurospora crassa* (NcDnfA, NcDnfB, NcDnfC1, NcDnfC2, and NcDnfD), *Aspergillus* *nidulans* (AnDnfA, AnDnfB, AnDnfC, and AnDnfD), and *Magnaporthe oryzae* (MoPde1, MoApt2, MoApt3, MoApt4, and MoApt5) using neighbor-joining method from MEGA 7 software. Values on clusters branches represent the results of bootstrap analysis. (B) Southern blot hybridization analysis of the indicated mutants using hygromycin DNA fragment (*HPH*) as a probe.
