# Supplementary material for: Flippases play specific but distinct roles in the development, pathogenicity, and secondary metabolism of Fusarium graminearum
Source: Mol Plant Pathol. 2020 Sep 2;21(10):1307–21. doi: 10.1111/mpp.12985 (PMC7488471; doi:10.1111/mpp.12985)
Supplement: Supplementary file 2 — FIGURE S2 Environmental stress responses of the flippase mutants in Fusarium graminearum. (a) Colonies of each strain growing on CM medium containing 0.01% SDS, 0.8 M NaCl, 12 mM H2O2, 0.5 mg/ml Congo red (CR) or 250 μg/ml calcofluor white (CFW). (b) Statistical analysis of mycelial growth inhibition due to the indicated stress‐inducing agents. Error bars represent SD from three replicates and the same letters on top of the bars indicate insignificant differences at p ≥ .05 [file MPP-21-1307-s002.docx]

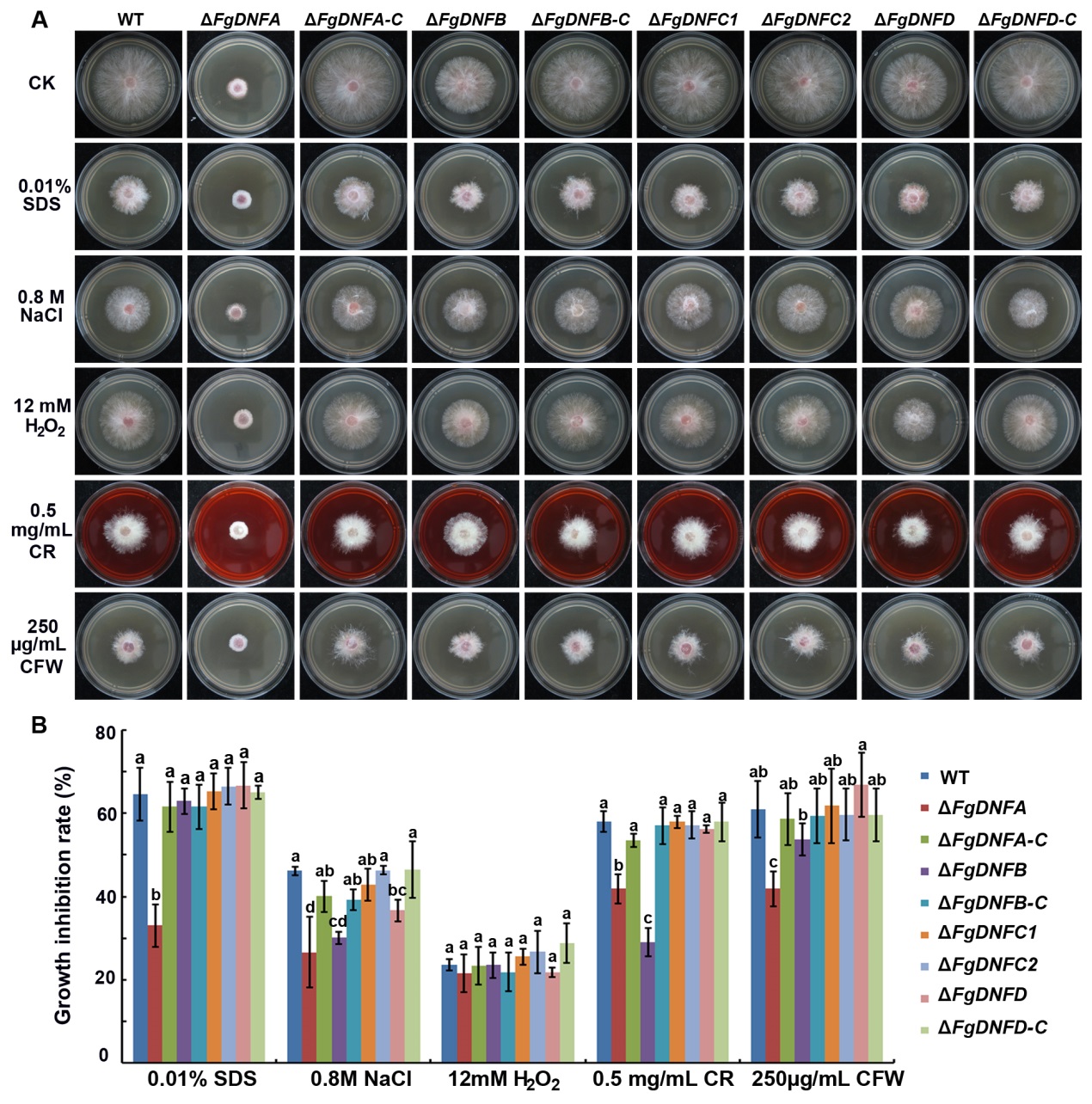


**Fig. S2 Environmental stress responses of the flippase mutants in *F. graminearum.***

(A) Colonies of each strain growing on CM media containing 0.01% SDS, 0.8M NaCl, 12mM H2O2, 0.5 mg/ml Congo red (CR) or 250μg/ml Calcofluor white (CFW). (B) Statistical analysis of mycelial growth inhibition due to the indicated stress-inducing agents. Error bars represent SD from three replicates, and same kind of letters on top of the bars indicate insignificant difference at *P* ≥ 0.05.
