# Supplementary material for: Flippases play specific but distinct roles in the development, pathogenicity, and secondary metabolism of Fusarium graminearum
Source: Mol Plant Pathol. 2020 Sep 2;21(10):1307–21. doi: 10.1111/mpp.12985 (PMC7488471; doi:10.1111/mpp.12985)
Supplement: Supplementary file 3 — FIGURE S3 Conidial germination rates of the flippase mutants. Fresh conidia were inoculated in CM liquid for 1, 2, 3, and 4 hr, and the germination of 50 conidia from the indicated strain was observed, respectively. The same letters on top of the bars indicate insignificant difference at p ≥ .05 [file MPP-21-1307-s003.docx]

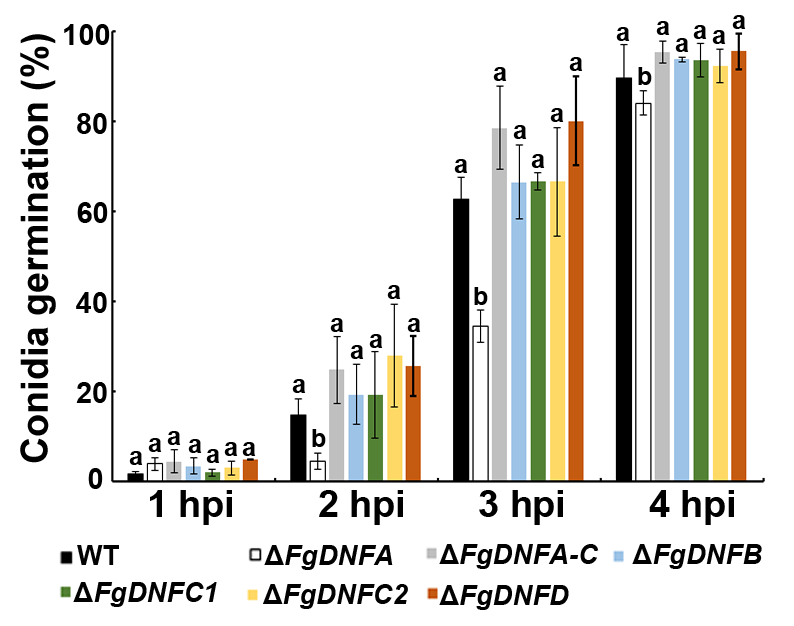


**Fig. S3 Conidia germination rates of the flippase mutants**

Fresh conidia were inoculated in CM liquid for 1 h, 2h, 3h, and 4h, and the germination of 50 conidia from the indicated strain was observed, respectively. Same kind of letters on top of the bars indicate insignificant difference at *P* ≥ 0.05.
