# Supplementary material for: Flippases play specific but distinct roles in the development, pathogenicity, and secondary metabolism of Fusarium graminearum
Source: Mol Plant Pathol. 2020 Sep 2;21(10):1307–21. doi: 10.1111/mpp.12985 (PMC7488471; doi:10.1111/mpp.12985)
Supplement: Supplementary file 4 — FIGURE S4 Protein structure of the different flippases in Fusarium graminearum. The structural representations of the five flippase proteins in F. graminearum are shown. Blue represents the transmembrane region, pink represents the low complexity region, and green stands indicates the coiled coil region [file MPP-21-1307-s004.docx]

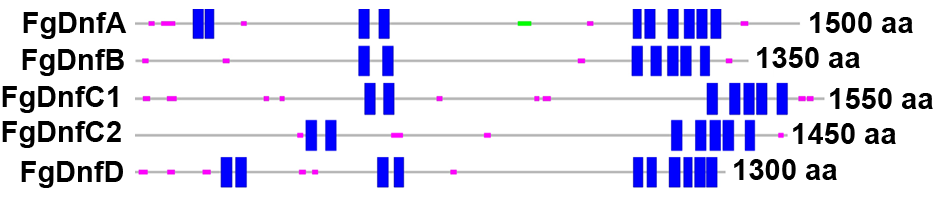


**Fig. S4 Protein structure of the different flippases in *F. graminearum***

The structural representations of the five flippase proteins in *F. graminearum* are shown. Blue represents transmembrane region; pink represents low complexity region and green stands for coiled coil region.
