# Supplementary material for: Flippases play specific but distinct roles in the development, pathogenicity, and secondary metabolism of Fusarium graminearum
Source: Mol Plant Pathol. 2020 Sep 2;21(10):1307–21. doi: 10.1111/mpp.12985 (PMC7488471; doi:10.1111/mpp.12985)
Supplement: Supplementary file 5 — FIGURE S5 Analyses of endocytosis in the flippase mutants of Fusarium graminearum. FM4‐64 dye internalization in the wild‐type, ΔFgDNFA, ΔFgDNFB, ΔFgDNFC1, ΔFgDNFC2, and ΔFgDNFD was observed after 5 and 30 min. Bar = 10 μm [file MPP-21-1307-s005.docx]

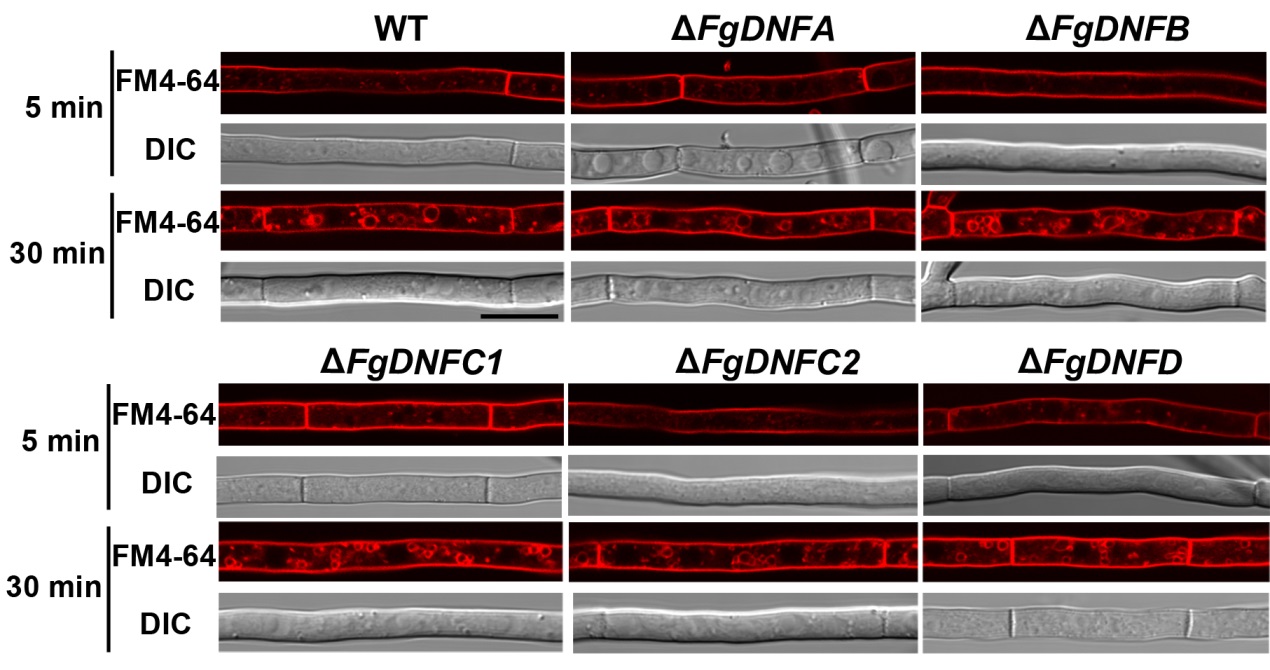


**Fig. S5 Analyses of endocytosis in the flippase mutants of *F. graminearum***

FM4-64 dye internalization in the wild-type, Δ*FgDNFA*, Δ*FgDNFB*, Δ*FgDNFC1*, Δ*FgDNFC2* and Δ*FgDNFD* was observed after 5, and 30 minutes. Bars=10 μm
