# Supplementary material for: Flippases play specific but distinct roles in the development, pathogenicity, and secondary metabolism of Fusarium graminearum
Source: Mol Plant Pathol. 2020 Sep 2;21(10):1307–21. doi: 10.1111/mpp.12985 (PMC7488471; doi:10.1111/mpp.12985)
Supplement: Supplementary file 6 — FIGURE S6 Relative gene expression levels of the five flippase genes in Fusarium graminearum. (a) The expression profiles of the five flippase genes at different stages of F. graminearum development: vegetative growth (potato dextrose agar), sexual induction for 3 days (S 3d) and 8 days (S 8d), and infection assay on wheat heads at 1, 2, and 3 days postinoculation (I 1d, I 2d, and I 3d). Each flippase gene was up‐regulated during the sexual process but down‐regulated during the infection process compared to the normal vegetative growth. (b) The relative expression levels of the flippase genes in the fungal mycelia grown on TBI compared to CM medium. Error bars represent SD from three replicates and the same letters on top of the bars indicate insignificant differences at p ≥ .05 [file MPP-21-1307-s006.docx]

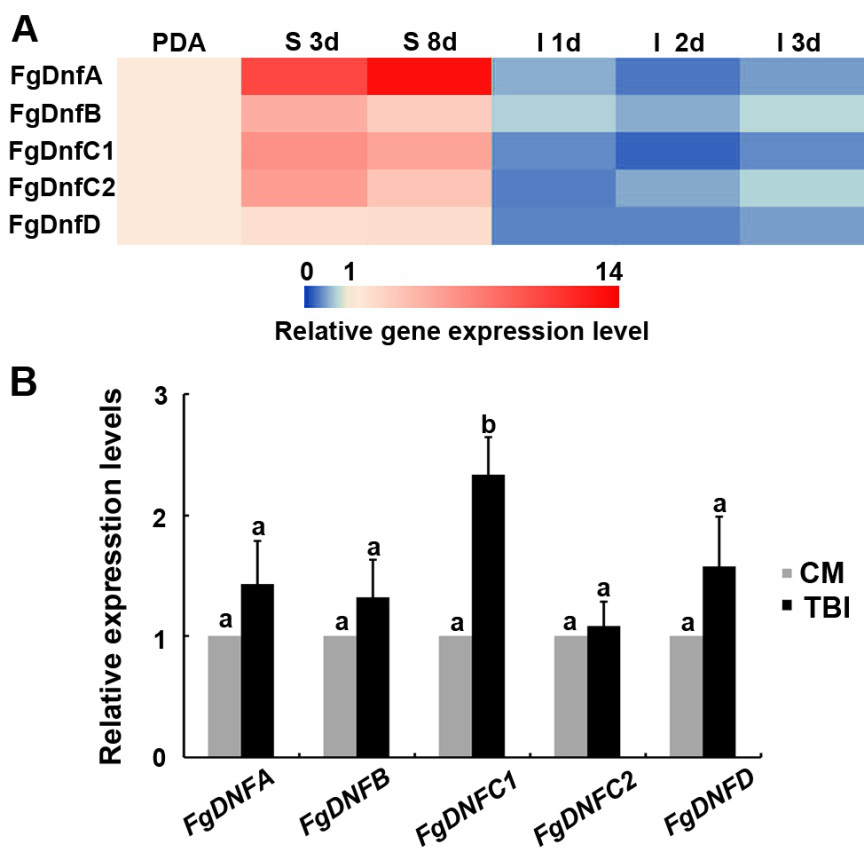


**Fig. S6 Relative gene expression levels of the five flippase genes in *F. graminearum*.**

(A) The expression profiles of the five flippase genes at different stages of *F. graminearum* development including vegetative growth (PDA media), sexual induction for 3 days (S 3d) and 8 days (S 8d), and infection assay on wheat heads at 1, 2 and 3 dpi (I 1d, I 2d, and I 3d). Each flippase gene was up-regulated during sexual process but down-regulated during infection process compared to the normal vegetative growth. (B) The relative expression levels of the flippase genes in the fungal mycelia grown on TBI compared to CM media. Error bars represent SD from three replicates, and same kind of letters on top of the bars indicate insignificant difference at P ≥ 0.05.
