# Supplementary material for: Flippases play specific but distinct roles in the development, pathogenicity, and secondary metabolism of Fusarium graminearum
Source: Mol Plant Pathol. 2020 Sep 2;21(10):1307–21. doi: 10.1111/mpp.12985 (PMC7488471; doi:10.1111/mpp.12985)
Supplement: Supplementary file 7 — FIGURE S7 Functional analyses of flippase double deletion mutants. (a) Colonies of the wild‐type strain, ΔFgDNFAC1, ΔFgDNFAC2, ΔFgDNFBC1, ΔFgDNFBC2, ΔFgDNFDC1, ΔFgDNFDC2, and ΔFgDNFDC1C2 mutants on complete medium (CM) and minimal medium (MM) plates after incubation for 3 days at 28 °C. (b) Fresh conidia from the indicated strains stained with CFW. Bar = 30 μm. (c) Perithecia (bar = 200 μm) and ascospores (bar = 10 μm) formation. (d) Pathogenicity of the indicated strains on wheat heads [file MPP-21-1307-s007.docx]

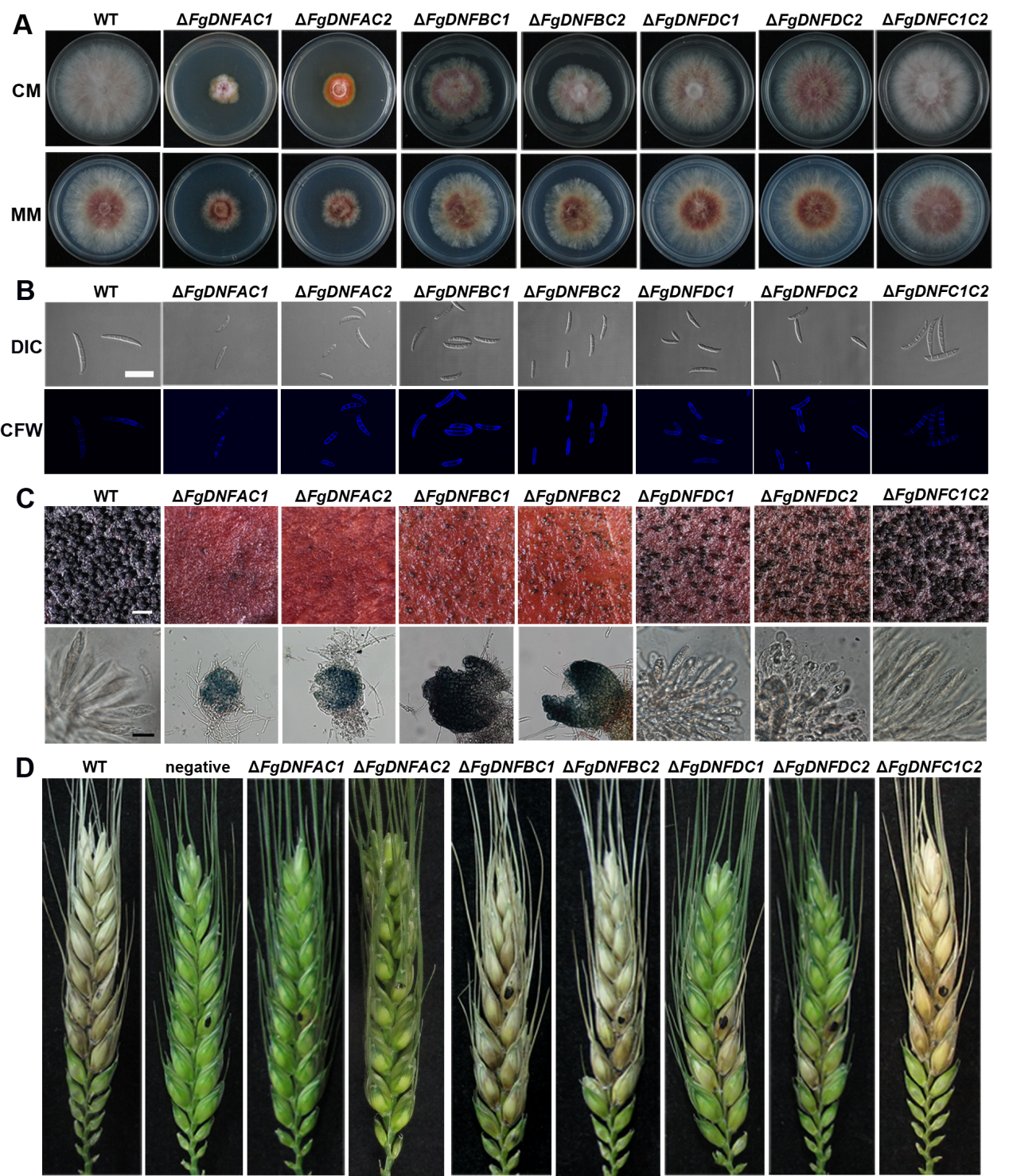


**Fig. S7 Functional analyses of flippase double deletion mutants**

(A) Colonies of the wild-type strain, Δ*FgDNFAC1*, Δ*FgDNFAC2*, Δ*FgDNFBC1*, Δ*FgDNFBC2*, Δ*FgDNFDC1*, Δ*FgDNFDC2* and Δ*FgDNFDC1C2* mutants on CM and MM plates after incubation for 3 days at 28 °C. (B) Fresh conidia from the indicated strains stained with CFW. Bar=30 μm. (C) Perithecia (Bar=200 μm) and ascospores (Bar=10 μm) formation. (D) Pathogenicity of the indicated strains on wheat heads.
