# Supplementary material for: Flippases play specific but distinct roles in the development, pathogenicity, and secondary metabolism of Fusarium graminearum
Source: Mol Plant Pathol. 2020 Sep 2;21(10):1307–21. doi: 10.1111/mpp.12985 (PMC7488471; doi:10.1111/mpp.12985)
Supplement: Supplementary file 9 — FIGURE S9 Cellular localization of NBD‐PC in flippase mutants of Fusarium graminearum. The wild‐type strain, flippase single and double gene deletion mutants were treated with NBD‐PC and observed under a confocal microscope. Bar = 10 μm [file MPP-21-1307-s009.docx]

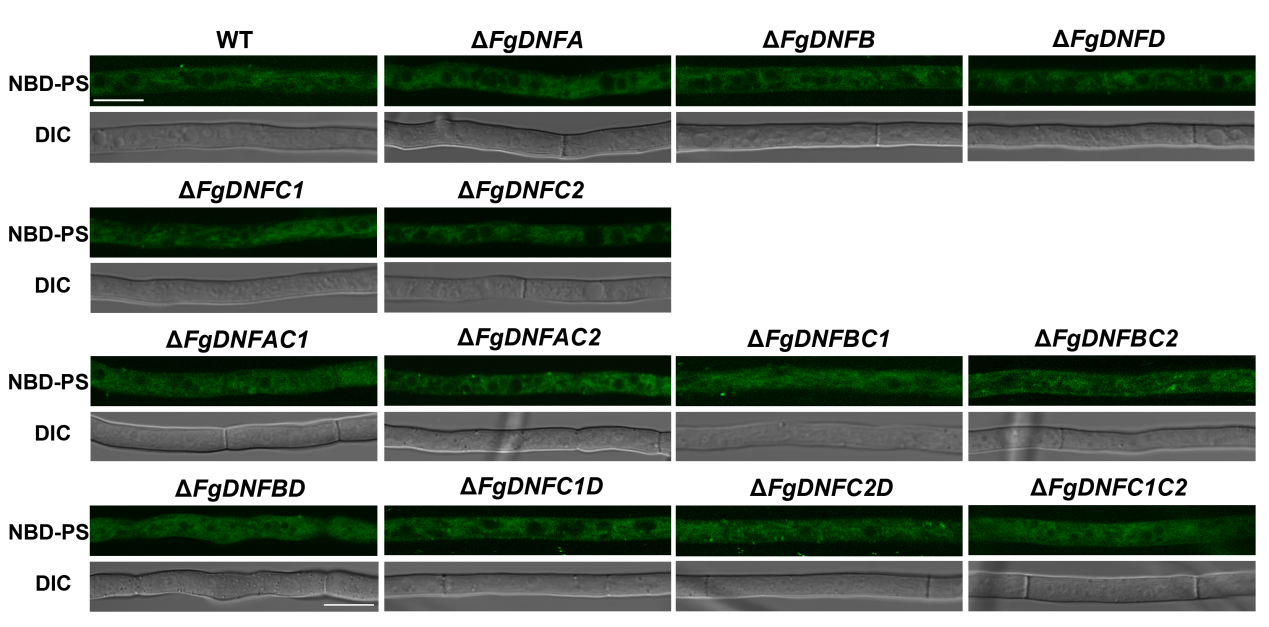


**Fig. S9 Cellular localization of NBD-PC in flippase mutants of *F. graminearum***

The wild-type strain, flippase single and double gene deletion mutants were treated with NBD-PC and observed under a confocal microscope. Bar=10 μm.
