# Supplementary material for: Flippases play specific but distinct roles in the development, pathogenicity, and secondary metabolism of Fusarium graminearum
Source: Mol Plant Pathol. 2020 Sep 2;21(10):1307–21. doi: 10.1111/mpp.12985 (PMC7488471; doi:10.1111/mpp.12985)
Supplement: Supplementary file 10 — TABLE S1 The primers used in this study [file MPP-21-1307-s010.docx]

Table S1. The primers used in this study

| Primer | Sequence | Relevant characteristics |
| --- | --- | --- |
| FgDNFA-up-F | AGCCGATAGATCGTGTCCAGT | PCR primers to amplify upstream fragment of *FgDNFA* for single gene deletion |
| FgDNFA -up-R | CATTCATTGTTGACCTCCACTAGCTCCACCCAACGCGATCTTTGAGTA |  |
| FgDNFA-d-F | GCAAAGGAATAGAGTAGATGCCGACCGTTCATGCCTGGCACCTTCT | PCR primers to amplify downstream fragment of *FgDNFA* for single gene deletion |
| FgDNFA-d-R | TTGCCTTCGTTGCGTGTTT |  |
| HPH-F | TGGAGCTAGTGGAGGTCAACA | PCR primers to amplify fragment of *HPH* |
| HPH-R | CGGTCGGCATCTACTCTATTC |  |
| FgDNFA-nest-F | CGACGCCGTTTATTTCCAA | PCR primers to amplify upstream- *HPH*-downstream fragment for deletion of *FgDNFA* |
| FgDNFA-nest-R | GGTGCGCCGTATAATTAGTTG |  |
| FgDNFA-id-F | TGTGGTAGCCATGGTCAAGAA | PCR primers for identification of  *FgDNFA* deletion transformants |
| FgDNFA-id-R | TTACCAGAACTTGAGCTGGGT |  |
| FgDNFA-GFP-id-F | AGGTCCAAGAAGATGCCGAA | PCR primers for identification  *FgDNFA-GFP* transformants |
| FgDNFA-GFP-id-R | TTGAAGAAGATGGTGCGCT |  |
| FgDNFA-Dup-F | TTGCTTACACAATTCCCCTG | PCR primers to amplify upstream fragment of *FgDNFA* for double genes deletion |
| FgDNFA-Dup-R | GAATGGAAATTGTAAGCGTTAATCTAGAAGAAACGTCACCCTCCAAAA |  |
| FgDNFA-Dd-F | CGCCTTCTTGACGAGTTCTTCTGACGCCGTCTTGCTGAGAGTATT | PCR primers to amplify upstream fragment of *FgDNFA* for double genes deletion |
| FgDNFA-Dd-R | TGCTGACGTAAAGTCGTTGCT |  |
| G418-F | TCTAGATTAACGCTTACAAT | PCR primers to amplify fragment of geneticin resistant gene (*NEO*) |
| G418-R | GCCCAATAGCAGCCAGTCC |  |
| FgDNFB-Dup-F | ACATGGCCATTCACCTGCAA | PCR primers to amplify upstream fragment of *FgDNFB* for double genes deletion |
| FgDNFB-Dup-R | GAATGGAAATTGTAAGCGTTAATCTAGATGTCCGGTATTGAAACCTCCT |  |
| FgDNFB-Dd-F | CGCCTTCTTGACGAGTTCTTCTGATCTGGCGCTCGGTATCCTA | PCR primers to amplify downstream fragment of *FgDNFB* for double genes deletion |
| FgDNFB-Dd-R | CGATCAAAAAAGAAGCGCGA |  |
| FgDNFBD-nest-F | AGCGAGTTAGCGAGCGTAGA | PCR primers to amplify upstream- *NEO* -downstream fragment for deletion of *FgDNFB* |
| FgDNFB-nest-R | CGCCCTGAAACTTGAGACAAA |  |
| FgDNFB-id-F | ATCGACAAGAGACTGCCATCA | PCR primers for identification of  *FgDNFB* deletion transformants |
| FgDNFB-id-R | ACTGTGTCATGTATAGGGCGA |  |
| FgDNFC1-up-F | ACATGCGTACGTTTCGAGG | PCR primers to amplify upstream fragment of *FgDNFC1* for single gene deletion |
| FgDNFC1-up-R | CATTCATTGTTGACCTCCACTAGCTCCACGGCGGACGATCATTATTG |  |
| FgDNFC1-d-F | GCAAAGGAATAGAGTAGATGCCGACCGTGATGAAGGTGCCTGAGCTGT | PCR primers to amplify downstream fragment of *FgDNFC1* for single gene deletion |
| FgDNFC1-d-R | ACATTCTCTGTGGAGAGGGGA |  |
| FgDNFC1-nest-F | TGGCGCTTGGTTAAGGTGTTA | PCR primers to amplify upstream- *HPH*-downstream fragment for deletion of *FgDNFC1* |
| FgDNFC1-nest-R | AATGACGGAGATTCCGGGTAT |  |
| FgDNFC1-id-F | TTCCAGACACGATCGACAAG | PCR primers for identification of  *FgDNFC1* deletion transformants |
| FgDNFC1-id-R | TCCTCATGTAATTCCATCGGC |  |
| FgDNFC1-GFP-id-F | TTGGACCTTATATCGTCCGAG | PCR primers for identification  *FgDNFC1-GFP* transformants |
| FgDNFC1-GFP-id-R | TTGAAGAAGATGGTGCGCT |  |
| FgDNFC1-Dup-F | ACATGCGTACGTTTCGAGG | PCR primers to amplify upstream fragment of *FgDNFC1* for double genes deletion |
| FgDNFC1-Dup-R | GAATGGAAATTGTAAGCGTTAATCTAGACGGCGGACGATCATTATTG |  |
| FgDNFC1-Dd-F | GCAAAGGAATAGAGTAGATGCCGACCGTGATGAAGGTGCCTGAGCTGT | PCR primers to amplify downstream fragment of *FgDNFC1* for double genes deletion |
| FgDNFC1-Dd-R | ACATTCTCTGTGGAGAGGGGA |  |
| FgDNFC2-up-F | ACCGATGAGATCAGGGTTTGA | PCR primers to amplify upstream fragment of *FgDNFC2* for single gene deletion |
| FgDNFC2-up-R | CATTCATTGTTGACCTCCACTAGCTCCATCGAAGGTTCAGGTCCCAAT |  |
| FgDNFC2-d-F | GCAAAGGAATAGAGTAGATGCCGACCGTGTTATCTGTATGGGGGTGTG | PCR primers to amplify downstream fragment of *FgDNFC2* for single gene deletion |
| FgDNFC2-d-R | AATACGACGGAGATCCGTATG |  |
| FgDNFC2-nest-F | GGGCAAGGGACTTTGTTCTAT | PCR primers to amplify upstream- *HPH*-downstream fragment for deletion of *FgDNFC2* |
| FgDNFC2-nest-R | GGCGATGAAGTCTATTAACGG |  |
| FgDNFC2-id-F | CGGGTGACATGATTGAACAGT | PCR primers for identification of  *FgDNFC2* deletion transformants |
| FgDNFC2-id-R | AGTTCCATCGTCCATGAACA |  |
| FgDNFC2-GFP-id-F | TGGTTCGCTTGGCTGTCTTT | PCR primers for identification  *FgDNFC2-GFP* transformants |
| FgDNFC2-GFP-id-R | TTGAAGAAGATGGTGCGCT |  |
| FgDNFC2-Dup-F | ACCGATGAGATCAGGGTTTGA | PCR primers to amplify upstream fragment of *FgDNFC2* for double genes deletion |
| FgDNFC2-Dup-R | GAATGGAAATTGTAAGCGTTAATCTAGATCGAAGGTTCAGGTCCCAAT |  |
| FgDNFC2-Dd-F | CGCCTTCTTGACGAGTTCTTCTGATGTTATCTGTATGGGGGTGTG | PCR primers to amplify downstream fragment of *FgDNFC2* for double genes deletion |
| FgDNFC2-Dd-R | AATACGACGGAGATCCGTATG |  |
| FgDNFD-UP-F | CCATGTCAGGCACAGATCAT | PCR primers to amplify upstream fragment of *FgDNFD* for single gene deletion |
| FgDNFD-UP-R | CATTCATTGTTGACCTCCACTAGCTCCATCGGAAGGGCGGTATTGTT |  |
| FgDNFD-d-F | GCAAAGGAATAGAGTAGATGCCGACCGGTCAAGCTCTTGGTCTGGCAT | PCR primers to amplify downstream fragment of *FgDNFD* for single gene deletion |
| FgDNFD-d-R | ACTTGCAAAGGCAAAACCCT |  |
| FgDNFD-nest-F | TCCAGCTCAATGAATTGTCG | PCR primers to amplify upstream- *HPH*-downstream fragment for deletion of *FgDNFD* |
| FgDNFD-nest-R | TAACAGTGGCGCCGTCTA |  |
| FgDNFD-id-F | ACGGCCAGTATCAAGAATTCG | PCR primers for identification of  *FgDNFD* deletion transformants |
| FgDNFD-id-R | ACATCGGCAGCTTGAATCAT |  |
| FgDNFD-GFP-id-F | TGATCCAGGGATTGTCACAGA | PCR primers for identification  *FgDNFD-GFP* transformants |
| FgDNFD-GFP-id-R | TTGAAGAAGATGGTGCGCT |  |
| FgDNFD-DUP-F | CCATGTCAGGCACAGATCAT | PCR primers to amplify upstream fragment of *FgDNFD* for double genes deletion |
| FgDNFD-DUP-R | GAATGGAAATTGTAAGCGTTAATCTAGATCGGAAGGGCGGTATTGTT |  |
| FgDNFD-Dd-F | CGCCTTCTTGACGAGTTCTTCTGAGTCAAGCTCTTGGTCTGGCAT | PCR primers to amplify downstream fragment of *FgDNFD* for double genes deletion |
| FgDNFD-Dd-R | ACTTGCAAAGGCAAAACCCT |  |
| Fg03541-GFP-id-F | CTTTCCCCAGTCTTCTTGAG | PCR primers for identification  *Fg03541-GFP* transformants |
| Fg03541-GFP-id-R | TTGAAGAAGATGGTGCGCT |  |
| Tri 1 qRT-F | TCCAGACTACGAAGTGCTA | QPCR primers of *FgTRI 1* |
| Tri 1 qRT-R | TCATCCTGTACCAATTCCAAT |  |
| Tri 4 qRT-F | ACCAGGTCCTCAGTCTTG | QPCR primers of *FgTRI 4* |
| T ri 4 qRT-R | TCGTTGTGCTTGCCATAG |  |
| Tri 5 qRT-F | TGAGGGATGTTGGATTGAGCAGTAC | QPCR primers of *FgTRI 5* |
| Tri 5 qRT-R | TGCTTCCGCTCATCAAACAGGT |  |
| Tri 6 qRT-F | GCTACTCAGAATGCCCTCAG | QPCR primers of *FgTRI 6* |
| Tri 6 qRT-R | CGCATGTTATCCACCCTGCTA |  |
| Tri 12 qRT-F | GCTGTAACTGTCCCCAGCAT | QPCR primers of *FgTRI 12* |
| Tri 12 qRT-R | GTGAAGTTGCGACCGTACTC |  |
| Tri 101 qRT-F | ATACCCTATGGCGATGTTTGAC | QPCR primers of *FgTRI 101* |
| Tri 101 qRT-F | CTGTCCGTTGACAGTGAGGAT |  |
| Hmr 1 qRT-F | GCCCGTCTTCAATCTATG | QPCR primers of *FgHmr 1* |
| Hmr 1 qRT-F | CGTCACCAGTAGTAGTCT |  |
| Tublin-F | GTCAGTGCGGTAACCAAATCGGT | QPCR primers of *Tublin* |
| Tublin-R | CTCAGAGGTGCCGTTGTAAACACC |  |
